# Discovery and Heterologous Expression of Functional 4-*O*-Dimethylallyl-l-tyrosine Synthases from Lichen-Forming Fungi

**DOI:** 10.1021/acs.jnatprod.4c00619

**Published:** 2024-09-10

**Authors:** Riccardo Iacovelli, Siqi He, Nika Sokolova, Iris Lokhorst, Maikel Borg, Peter Fodran, Kristina Haslinger

**Affiliations:** Department of Chemical and Pharmaceutical Biology, Groningen Research Institute of Pharmacy, University of Groningen, 9713 AV Groningen, The Netherlands

## Abstract

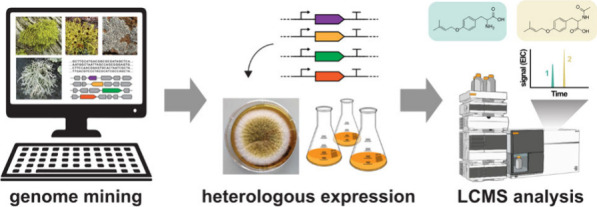

Fungal aromatic prenyltransferases are a family of biosynthetic enzymes that catalyze the prenylation of a range of aromatic substrates during the biosynthesis of bioactive indole alkaloids, diketopiperazines, and meroterpenoids. Their broad substrate scope and soluble nature make these enzymes particularly adept for applications in biocatalysis; for example, the enzymatic derivatization of aromatic drugs improves their bioactivity. Here, we investigated four putative aromatic prenyltransferases from lichen-forming fungi, an underexplored group of organisms that produce more than 1,000 unique metabolites. We successfully expressed two enzymes, annotated as dimethylallyltryptophan synthases, from two lichen species in the heterologous host *A. oryzae*. Based on their in vivo activity, we hypothesize that these enzymes are in fact 4-*O*-dimethylallyl-l-tyrosine synthases. Our extensive bioinformatic analysis further confirmed that these and related lichen aromatic prenyltransferases are likely not active on indoles but rather on aromatic polyketides and phenylpropanoids, major metabolites in lichens. Overall, our work provides new insights into fungal aromatic prenyltransferases at the family level and enables future efforts aimed at identifying new candidates for biocatalytic transformations of aromatic compounds.

Fungi are proficient producers of secondary metabolites: specialized molecules that are not strictly required for growth or survival, but that play important roles in sexual development, communication and competition with other organisms, and defense from environmental stressors.^1^ In particular, lichen-forming fungi—organisms living in symbiotic associations with green algae or cyanobacteria in nature—are prolific producers of secondary metabolites, notably bioactive aromatic polyketides and natural dyes.^2−5^ Lichens are relatively underexplored because of their complex biology, as well as the technical challenges around their cultivation in the laboratory.^6^ Thanks to advances in DNA sequencing technologies and bioinformatics, it has recently become easier to obtain high-quality genome sequences from lichen-forming fungi and thus gauge their biosynthetic capabilities.^7−9^ A major challenge, however, remains the heterologous expression of genes originating from these organisms, hampering the linking of metabolites to biosynthetic genes.^10,11^ To date, there are only three studies reporting the successful expression of lichen genes in heterologous hosts: the orotidine-5′-decarboxylase-encoding *pyrG* gene from *Solorina crocea*(12) and two polyketide synthase-encoding genes from *Pseudevernia furfuracea*(13) and *Stereocaulon alpinum.*(14) Despite this challenge, we set out to explore the biosynthetic potential of lichen-forming fungi experimentally and thus analyzed their predicted biosynthetic gene clusters in silico. Surprisingly, we found that 67 genes were annotated as dimethylallyltryptophan synthases (DMATS), biosynthetic enzymes belonging to the superfamily of ABBA-type aromatic prenyltransferases (PTs).^15−17^ DMATSs are involved in the biosynthesis of indole alkaloids—compounds with a broad range of biological activities in animals, most notably on the nervous and circulatory systems.^18^ However, no indole alkaloids have ever been reported in extracts of lichens.^19^

Intrigued by this finding, we selected 4 putative DMATS-type PTs from lichen-forming fungi and overexpressed them in the heterologous host *Aspergillus oryzae* for in vivo characterization. We furthermore carried out an extensive bioinformatic analysis to investigate sequence-function relationships in this important family of enzymes. The combined results revealed that the prenyltransferases from lichen-forming fungi have a distinct substrate scope that sets them apart from “prototype” DMATS, with potential implications in the discovery of new biocatalysts and new bioactive compounds.

## Results and Discussion

Based on our previous studies with the wolf lichen *Letharia lupina*,^20^ we analyzed the biosynthetic gene clusters (BGCs) in its recently published genome^7^ with the antiSMASH webtool.^21,22^ Among the expected high number of polyketide-producing BGCs (21 out of 42, in total), we also noticed 2 gene clusters annotated as indole-producing, with DMATS as the core biosynthetic enzyme. Intrigued by this observation, we analyzed all published lichen genomes for the presence of genes annotated as putative DMATS and identified 67 such genes. When further analyzing their genomic context by antiSMASH and gggenomes,^23^ two genomic regions struck us as interesting: region AS-522.1 in the genome of *Acarospora strigata* CBS 132363, annotated to encode a didomain DMATS-cytochrome P450 enzyme; and region RI-146.1 in the genome of *Ramalina intermedia* YAF0013, annotated to encode a didomain halogenase-acyltransferase enzyme in the vicinity of the DMATS-encoding gene (Table S1, Figure S1). In addition to these two intriguing genes, we also chose the putative DMATS-encoding genes KAF6225851.1 of *L. lupina* and its close relative KAF6239039.1 from *L. columbiana* for further characterization by heterologous expression in *Aspergillus oryzae* NSAR1.^24^ When cloning the target gene from the genomic DNA of *A. strigata*, we noticed that all clones were missing 28 nucleotides in the predicted exon 2, which results in a frameshift shortening the open reading frame to 1,358 nucleotides. This is the expected size for a DMATS-encoding gene, and we therefore concluded that the gene was in fact not coding for a didomain enzyme, but for a canonical DMATS-type PT. Thus, we proceeded with the expression of the four DMATS coding sequences.

### Heterologous Expression of Two Lichen DMATS in *A. oryzae* Yields 4-*O*-Dimethylallyl-l-tyrosine and Its N-Acetyl Derivative

**Figure 1:**
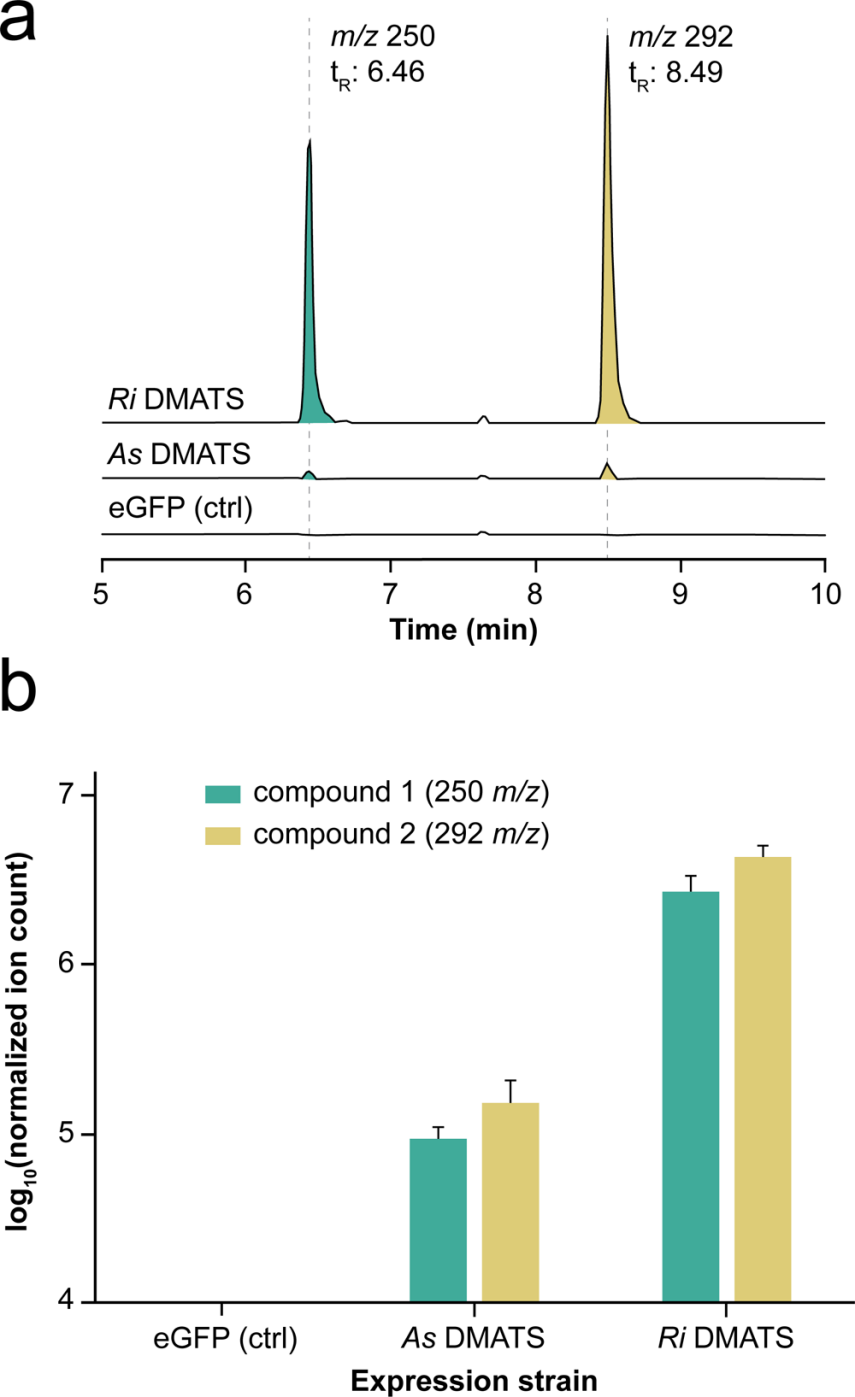
LC-MS-based detection of two new peaks in the extracts of DMATS expression strains. (a) Extracted ion chromatograms of fungal extracts for *m*/*z* 250 (**1**) and *m*/*z* 292 (**2**) (±0.2 Da). (b) Peak areas (log_10_ scale) of **1** and **2** in fungal extracts. The minimum value for the *y* axis is equal to a signal intensity of 1 × 10^4^, which corresponds to baseline levels. The values were normalized for the internal standard caffeine. Bars represent mean ± SD (*n* = 4). Only one control trace is shown.

We first obtained the synthetic gene encoding *Ri* DMATS, while the sequences encoding *As* DMATS, *Ll* DMATS and *Lc* DMATS were amplified directly from genomic DNA of *A. strigata* CBS 132363 and wild isolates of *L. lupina* and *L. columbiana* collected for a previous study.^20^ All genes of interest were cloned into the pTYargB or pTYadeA vectors^25^ for expression in the host *A. oryzae* NSAR1. Control strains transformed with the pTY plasmids carrying the eGFP gene were included to control for any variation in the metabolic profile caused by the presence of the plasmids themselves, and to visually confirm successful induction of the *amyB* cassette resulting in fluorescent mycelia. The transformed strains were first grown for 5 days on maltose-containing agar (inducing medium), followed by extraction of secondary metabolites from both mycelium and agar. HPLC-MS-DAD analysis of the extracts did not reveal the production of any new metabolites in the *Ll* DMATS and *Lc* DMATS expression strains compared to the controls. However, both *Ri* DMATS and *As* DMATS expression led to the production of two new compounds (**1** and **2**) with *m*/*z* 250 and 292, respectively (Figures 1a and S2), albeit at different levels. Indeed, the concentrations of **1** and **2** in the extracts from the *Ri* DMATS expression strain were ∼30 higher compared to those in the extracts of the *As* DMATS expression strain (Figure 1b). As neither *m*/*z* values matched the expected value for dimethylallyltryptophan (*m*/*z* = 273 [M + H]^+^), we subjected the same samples to HRMS-MS to attempt compound identification. Compound 1 was predicted to have the molecular formula C_14_H_19_NO_3_ based on *m*/*z* 250.1438 [M + H]^+^ (calcd for C_14_H_20_NO_3_^+^, 250.1443), consistent with the attachment of a dimethylallyl moiety to the amino acid tyrosine. Indeed, MS2 data showed the typical fragmentation pattern of tyrosine (Table S2). Compound **2** was predicted to have the molecular formula C_16_H_21_NO_4_ based on *m*/*z* 292.1547 [M + H]^+^ (calcd for C_16_H_22_NO_4_^+^, 292.1549), corresponding to prenylated acetyl-tyrosine. Once again, MS2 data confirmed the hypothesis showing the expected fragmentation pattern of N-acetyl-tyrosine, a naturally occurring derivative of the amino acid (Table S2). At this stage, we cannot confirm whether the DMATS enzymes are indeed able to attach the dimethylallyl moiety to this derivative, or whether N-acetylation occurs postprenylation. It is possible that native enzymes from *A. oryzae* acetylate compound **1** in response to its accumulation in the cells.

**Figure 2:**
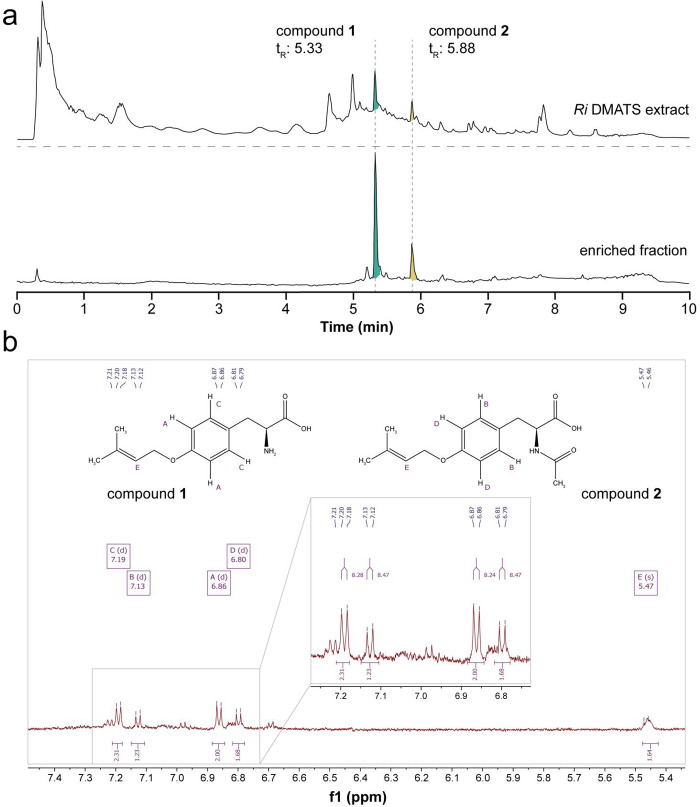
Structural characterization of compounds **1** and **2**. (a) Total ion chromatograms of total culture extract from *Ri* DMATS expression strain and enriched fraction containing compounds **1** and **2** obtained via semipreparative HPLC. Retention times are different compared to Figure 1 because a shorter analysis method was used here. (b) Chemical shifts of aromatic protons from the ^1^H NMR spectrum (600 MHz) of the enriched fraction (Figure S3), assigned based on ^1^H NMR spectra of reference compounds (Figures S6 and S8). The 4 major doublets indicate that the aromatic rings of **1** and **2** are free of substitutions, consistent with prenylation occurring on the 4-OH group of the substrates. The chemical shift of the prenyl proton is also observed.

To determine the site of prenylation, we set out to purify 1 and 2 from the total fungal extract of the *Ri* DMATS expression strain and perform ^1^H NMR. Because the compounds are only produced on solid media and in limited amounts, we first attempted purification via semipreparative HPLC to be able to work with smaller culture and solvent volumes. By applying one round of semipreparative HPLC on an RP-C18 column, we obtained a relatively pure fraction containing both compounds 1 and 2 from a total volume of 1 L of cultivation media and mycelia (Figure 2a). Given the structural similarity of the two compounds, we hypothesized that their ^1^H NMR spectra would largely overlap, and therefore we analyzed the enriched fraction without further purification. The resulting spectrum (Figures 2b and S3) shows a clear para–disubstitution pattern, indicating that the site of prenylation must be the 4-OH group of the tyrosine backbone. Informed by this observation, we synthesized 4-O-dimethyllalyltyrosine and its N-acetylated derivative (Schemes S1–S2 and S3–S4, respectively) and analyzed them via HPLC-MS-DAD, HRMS-MS, and ^1^H and ^13^C NMR (Figures S4–S11). The results matched the spectra measured for 1 and 2 from the fungal extract, confirming the chemical identity of the DMATS products.

### Sequence Similarity Network Analysis Suggests That Lichen DMATS-Type PTs Are Mainly Active on Polyketide and Phenylpropanoid Compounds but Not on Indoles

**Figure 3:**
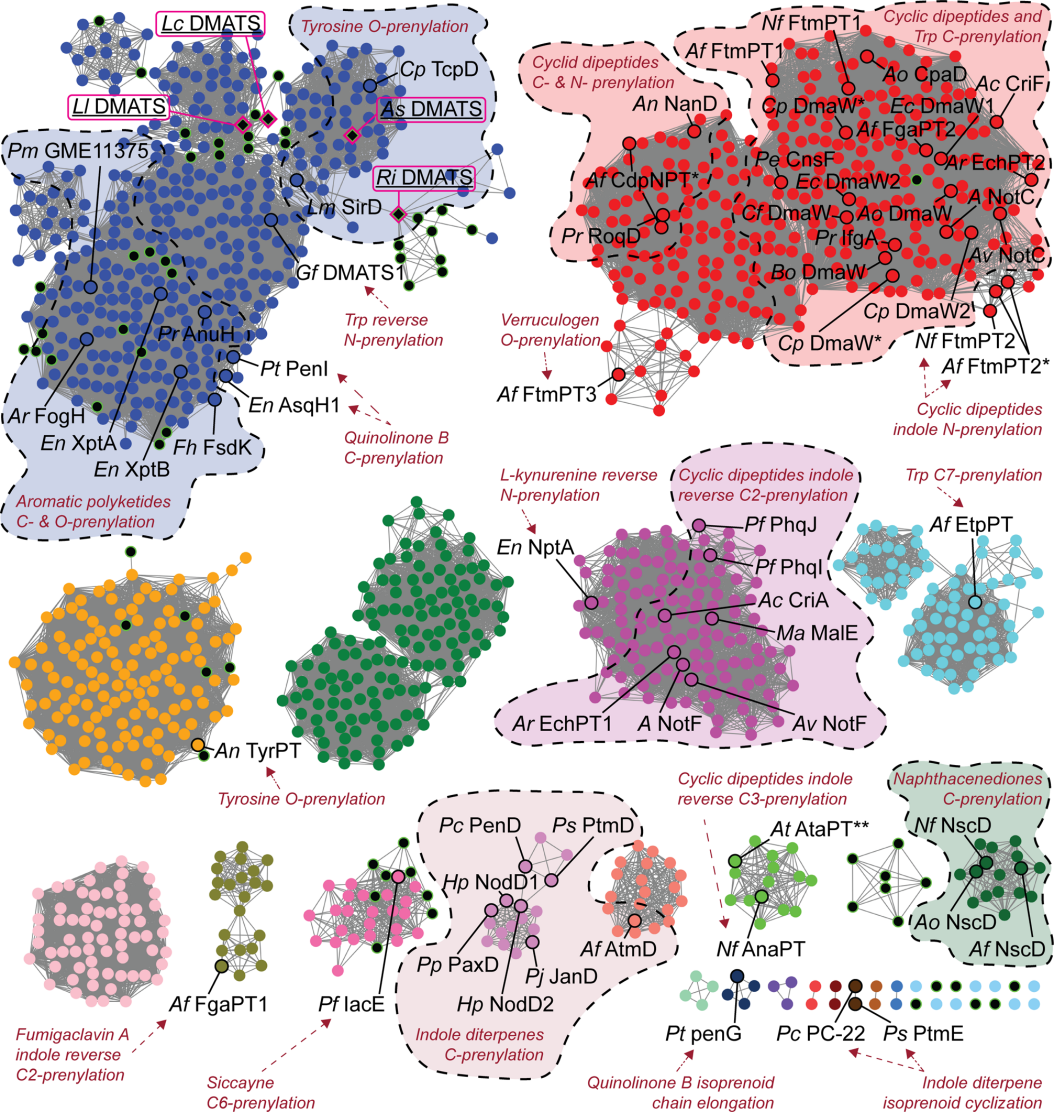
SSN of fungal DMATS-type PTs. The organic layout from yFiles was applied. Edge selection cutoff: sequence ID% > 34.2. The different clusters are colored based on the default color scheme of EFI-EST. Lichen enzymes are colored in black with green borders, regardless of their location within the network. The 4 enzymes subject of this work are highlighted in pink boxes and with underlined labels. Enzymes with known substrate specificity and type of reaction catalyzed (indicated in red) are circled in black and labeled (organism + enzyme name). For visualization purposes, dashed lines encircle groups of enzymes that catalyze the same type of reaction, although it should be stated that this notation only refers to characterized enzymes labeled in black. *Enzymes from the same species but different strains. **For simplicity, no annotation on substrate specificity/reaction was included for At AtaPT because of its broad donor (DMAPP, GPP, FPP) and acceptor (peptides, flavonoids, aromatics) substrate scope.^39^

To gain further insights into the sequence similarity of lichen PTs annotated as DMATS to other fungal DMATS, we performed pairwise sequence comparisons and constructed a sequence similarity network. For that, we first collected all sequences of fungal DMATS-type PTs from the InterPro database (IPR012148).^26^ We then manually extracted the sequences of all DMATS-type PT enzymes from 38 lichen genomes available publicly and appended them to the InterPro set. After removing duplicate sequences, we obtained a curated set of 1,446 entries. Finally, we used the EFI-Enzyme Similarity Tool (EST)^27^ to construct the sequence similarity network shown in Figure 3. We manually inspected the network and annotated all the nodes corresponding to biochemically characterized entries from literature and the SwissProt database^28,29^ (black circles). For these, both the substrate and type of prenylation reaction catalyzed were either determined experimentally or deduced from the chemical structure of the natural product synthesized by their BGC. What we observed is that, generally, enzymes with similar activity cluster together. This is particularly clear for the largest cluster (in red) which contains some of the best studied prototypical DMATS enzymes. These are responsible for C-4, C-5 and C-7-prenylation of tryptophan or tryptophan-containing cyclic dipeptides. The indole diterpene PTs (light pink), the cyclic dipeptide reverse C-2-PTs (fuchsia), and the aromatic polyketide PTs (part of the blue cluster) further exemplify this trend. Furthermore, we carried out a phylogenetic analysis of the biochemically characterized DMATS which revealed that there is a clear distinction between prototype DMATS, PTs active on larger indoles (cyclic peptides and indole diterpenes), and PTs active on non-indole aromatics such as tyrosine, phenylpropanoids, quinolines and large cyclic polyketides (Figure S12). This was also described in a recent publication on a tyrosine O-PT from an edible mushroom.^30^ Unsurprisingly, *As* DMATS and *Ri* DMATS cluster closely with the two well characterized 4-*O*-tyrosine PTs *Lm* SirD and *Cp* TcpD. Interestingly, the majority of DMATS from lichen-forming fungi (Figure 3, black nodes with green outline) reside within this cluster as well. Many of them are found in the vicinity of tyrosine-PTs—including the two enzymes from *Letharia* that we cloned—while the remaining ones are located within the aromatic polyketide PTs subcluster. Two PTs active on quinolinone B, a heterocyclic compound derived from anthranilic acid and *O*-methyl-tyrosine, are also present in this group. Six lichen PTs appear as singletons, while 7 others form an isolated cluster. The remaining are found in two poorly annotated clusters that contain only one reviewed entry each: the 4-*O*-dimethylallyl-l-tyrosine synthase *An* tyrPT^31^ (orange) and a PT active on siccayne (light pink), a phenolic compound likely derived from tyrosine or phenylalanine.^32^ These observations strongly suggest that lichen DMATS-type PTs might be mainly active on polyketide compounds and aromatic scaffolds derived from tyrosine and/or phenylalanine, but not on indole-containing scaffolds. In fact, aromatic polyketides (such as depsides, depsidones, chromones, xanthones, and anthraquinones), as well as shikimate-derived phenylpropanoids (terphenylquinolones and pulvinic acid derivatives), are the most abundant natural products isolated from lichen tissues.^4,11,33^ In contrast, nitrogen-containing secondary metabolites in lichens are rare, and indole alkaloids have not been isolated from lichens thus far. This is attributed to the fact that nitrogen is a limiting nutrient for lichen-forming fungi and therefore mostly restricted to primary metabolism.^19^ Indeed, our SSN analysis shows that only one out of 67 lichen PTs—from the organism *Usnea florida*—localizes in the prototype DMATS cluster. Interestingly, the corresponding BGC as predicted by fungiSMASH shows a high similarity with the ergotamine cluster from *Claviceps purpurea* (Figure S13)^34^ retrieved from the MIBiG database.^35^ Multiple sequence alignment (MSA) also showed that *Uf* DMATS shares the same catalytic residues of prototype tryptophan DMATS (Figure S14). Thus, further characterization of the BGC is of great interest as it might lead to the discovery of the first ergot alkaloid biosynthetic pathway in a lichen-forming fungus.

### Docking and MSA Studies Show Conserved Active Site Architecture and Substrate Binding in Putative Lichen 4-*O*-Dimethylallyl-l-tyrosine Synthases

**Figure 4:**
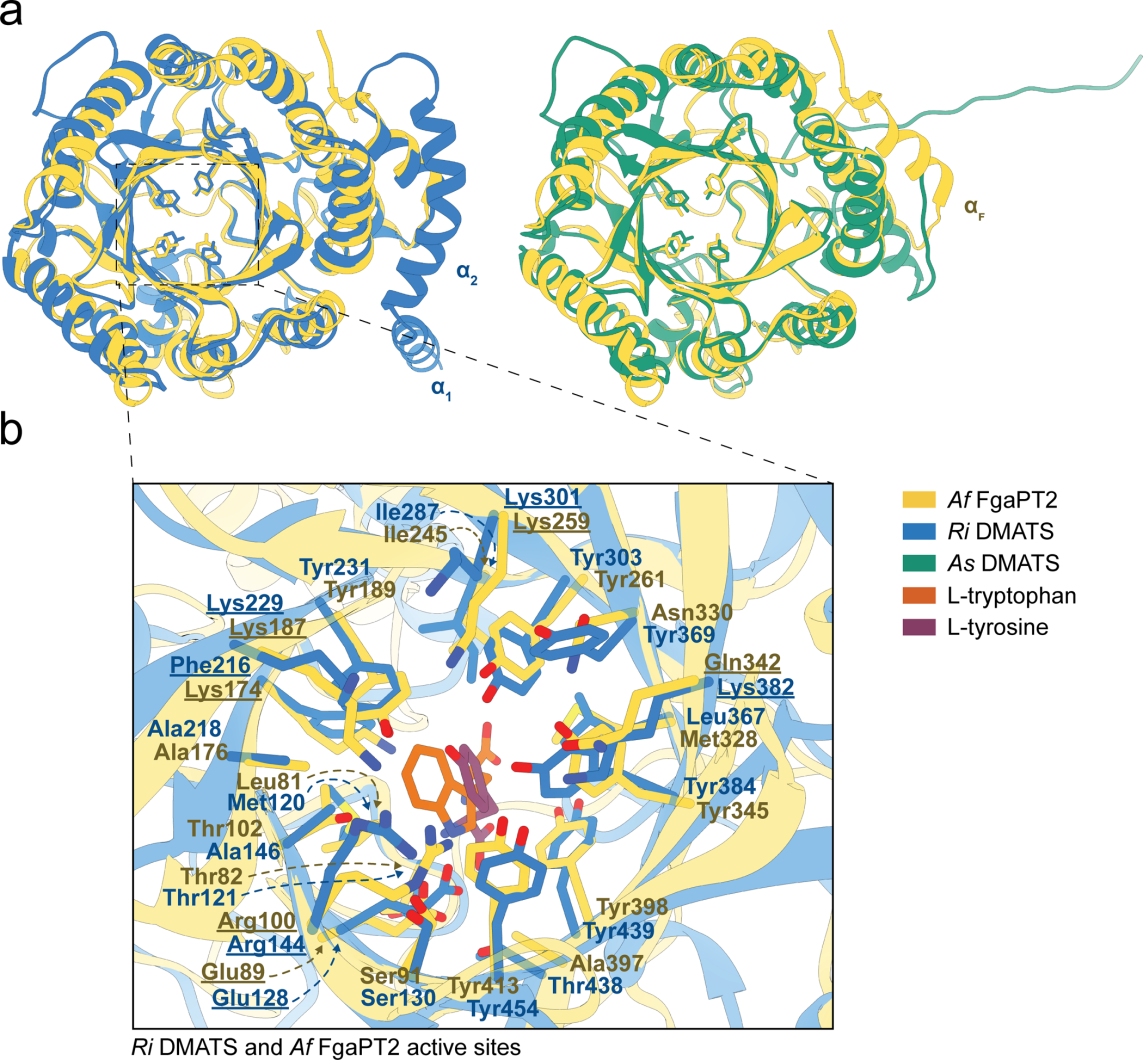
Comparison of *Ri* DMATS and *As* DMATS models with the crystal structure of *Af* FgaPT2 (PDB ID: 3I4X). (a) Superimposition of the structures shows that the overall ABBA fold and the central β-barrel where the tyrosine shield and active site are located are well conserved in the lichen DMATS. Two additional α-helices can be observed at the N-terminus of *Ri* DMATS, though these are predicted with low confidence (Figure S15). In the corresponding region, *As* DMATS is instead missing the N-terminal α-helix present in FgaPT2, and an additional, large, unstructured loop is predicted at its C-terminus (also with low confidence, Figure S15). (b) Active site comparison of *Ri* DMATS and *Af* FgaPT2. All amino acids within a 5 Å radius from the substrates are displayed as sticks colored by element: oxygen, red; nitrogen, dark blue; sulfur, light yellow; phosphate, orange; carbon, as indicated in the legend. The binding pockets show a similar architecture and substrate positioning. Key catalytic residues in FgaPT2 and corresponding residues in *Ri* DMATS are underlined. The structures were visualized using UCSF Chimera and aligned with the Matchmaker tool.

It is well known that DMATS are promiscuous enzymes and some of them can prenylate a remarkable range of aromatic substrates in vitro (e.g., tyrosine, tryptophan, cyclic peptides, lactones, quinolines, and even plant flavonoids).^36−39^ Though less promiscuous, the tyrosine-PTs *Lm* SirD and *An* TyrPT also show an extended substrate scope which includes tryptophan and its derivatives. Furthermore, these enzymes can catalyze prenylation at different positions—and atoms—on the aromatic ring.^31,40,41^ Although our experiments were only performed in vivo, we found it surprising that both *As* DMATS and *Ri* DMATS expression strains only produced 4-*O*-dimethylallyl-l-tyrosine and its N-acetylated derivative. Therefore, we used bioinformatic tools to generate structural models of the enzymes and investigate their active site architecture. First, we used AlphaFold^42^ to build models for both *As* and *Ri* DMATS and superimposed them to the crystal structure of *Af* FgaPT2^15^ to inspect the general structural features (Figure 4a). The overall ABBA fold and central β-barrel where the reaction chamber is located are very well conserved in the lichen enzymes. The tyrosine shield—a set of four Tyr residues that play a major role in catalysis^17^—also perfectly overlaps with that of FgaPT2. The only differences are observable at the termini of the proteins. At the N-terminus, *Ri* DMATS possesses two extra α-helices (α_1_ and α_2_) compared to FgaPT2, while the N-terminus of *As* DMATS is even shorter than that of FgaPT2, with a short unstructured region in place of helix α_F_. At the C-terminus, the model of *As* DMATS shows a relatively long unstructured region, which is absent in both *Ri* DMATS and FgaPT2. Intrigued by these differences, we also retrieved the structural models of the other 4-*O*-dimethylallyl-l-tyrosine synthases *Lm* SirD, *Cp* TcpD, and *An* TyrPT from the AlphaFold database^43^ and observed that all of them possess additional α-helices at their N-termini (Figure S15). It should be noted that in all cases the structures in this N-terminal region are predicted at very low confidence levels, thus we refrain from formulating any hypothesis on their putative function at this stage.

Next, we decided to have a deeper look into the active site architecture and possible binding mechanism of the lichen DMATS with the substrate l-tyrosine. Given that *As* DMATS and *Ri* DMATS possess the same overall structure and active site residues—and the fact that we detected higher product concentrations with *Ri* DMATS in vivo—we focused only on the latter. We first performed rigid molecular docking with the substrates l-tyrosine and DMAPP, followed by a 20 ns MD simulation to achieve reliable energy minimization and “stabilize” the complex. We then superimposed the resulting Tyr-bound structure (DMAPP was hidden for visualization purposes) with the crystal structure of FgaPT2 in complex with its substrate L-tryptophan.^15^ The results are shown in Figure 4b. Unsurprisingly, the overall architecture and the position of the key catalytic residues are well conserved. This is the case for Glu89 (Glu128 in *Ri* DMATS) which interacts with the indole –NH– in FgaPT2 and other prototype DMATS enzymes.^17^ It appears that a similar interaction is retained in *Ri* DMATS, where Glu128 interacts with the α-amino group of l-tyrosine. The DMAPP-binding residues Arg100 (Arg144), Lys187 (Lys229), and Lys259 (Lys301) are also conserved and show the same orientation in the structural models. On the other hand, the key residue Lys174, which acts as catalytic base to deprotonate the intermediate arenium ion in FgaPT2,^15^ is replaced by Phe216 in *Ri* DMATS. The aromatic ring of this residue is likely involved in a π–π interaction with the aromatic side chain of the substrate, rather than in catalysis. Indeed, a mutant variant of FgaPT2 where Lys174 was replaced by phenylalanine showed much higher specificity toward l-tyrosine, and its activity toward L-tryptophan was almost abolished.^44^ Another key difference is Gln343—involved in binding and positioning of the prenyl donor DMAPP in FgaPT2^15^—which is replaced by Lys382 in *Ri* DMATS. Both these differences are shared by other 4-*O*-dimethylallyl-l-tyrosine synthases (Figure S14), indicating conserved substrate binding modes and catalytic mechanisms. It was recently proposed that *Lm* SirD utilizes the conserved Glu residue (Glu89 in FgaPT2) or a water molecule as catalytic base,^17^ but our docking model shows that Lys382 might also play a crucial role for 4-O prenylation. In fact, its side chain is positioned in the vicinity of the 4-OH group of l-tyrosine. When we also display DMAPP in the pocket (Figure S16), it appears clear that the 4-OH group of tyrosine is in a favorable position for the nucleophilic attack onto the dimethylallyl cation. In further support of our hypothesis that Lys382 of *Ri* DMATS has a critical role for 4-O prenylation, the above-mentioned FgaPT2 K174F variant was indeed active on l-tyrosine, but prenylation would occur at the C3 position,^44^ which in our superimposition almost overlaps with the C4 of l-tryptophan (Figure 4b). Engineering a double mutant bearing both K174F and Q343K mutations could provide further evidence to validate our hypothesis. Interestingly, when looking at the sequences of fungal aromatic prenyltransferases from other groups than prototype DMATS, we observed that Gln343 is often replaced by lysine, but also arginine residues (and aspartate in one case) (Figure S14). We also noticed that Lys174 in these sequences is replaced by other residues which are conserved across enzymes that cluster together (Figure S14). As discussed for *Ri* DMATS, all 4-*O*-dimethylallyl-l-tyrosine synthases possess a conserved residue of phenylalanine; aromatic polyketide PTs and cyclic peptide PTs generally have small hydrophobic residues (Gly, Val, Ala, Leu); indole diterpene PTs have methionine residues; and cyclic dipeptide reverse C2-PTs have alanine residues. Given that these are all nonpolar residues, they are likely not directly involved in catalysis but rather in substrate positioning. The positively charged residues replacing Gln343, instead, might act as catalytic base to deprotonate the reaction intermediates. Interestingly, some indole diterpene PTs and the naphthacenedione PTs are the only ones that have different residues in place of the conserved Glu89 (Figure S14), which might at least in part have to do with the large size of their substrates.

## Conclusions

In this work, we report the successful expression of two functional DMATS-type PTs in a heterologous host, which allowed us to identify their 4-*O*-dimethylallyl-l-tyrosine synthase activity. *As* DMATS and *Ri* DMATS are the first enzymes from this family to be described in lichen-forming fungi. Furthermore, we performed SSN, MSA, and phylogenetic analyses which show that all lichen DMATS-type PTs but one are related to known TyrPTs and other aromatic PTs active on polyketides and phenylpropanoids. These are the major metabolites of lichen-forming fungi, which instead do not produce many nitrogen-containing secondary metabolites and are not known to make indole alkaloids. Thus, it is plausible that lichen-forming fungi evolved or maintained only PTs that can diversify non-indole aromatics for enhanced bioactivity. Lastly, we used molecular modeling and docking to investigate the active site architecture of *Ri* DMATS, which revealed that it is shared with known TyrPTs such as *Lm* SirD,^40^ and likely conserved among all TyrPTs. This suggests that the reaction mechanism is also conserved, though it remains yet to be elucidated experimentally. Generally, it is difficult to accurately predict substrate specificity, prenylation site, and the type of reaction catalyzed by a DMATS-type PT. This is because many factors such as substrate positioning, orientation of catalytic residues, and overall active site architecture, are at play. Nevertheless, we show that by using a combination of bioinformatic tools, it is possible to classify DMATS-type PTs in discrete groups that are active on substrates of the same chemical family.

Since many drugs and drug leads bear aromatic polyketides and phenylpropanoids scaffolds,^45,46^ we believe that further exploring lichen-forming fungi might lead to the discovery of DMATS-type PTs with desirable substrate scopes. These could readily be applied as biocatalysts or in combinatorial pathway engineering, to diversify or functionalize aromatic compounds and improve their biological and pharmacological activities.^36,38^ Ultimately, our findings may facilitate prioritization of interesting targets in future efforts aimed at identifying aromatic DMATS-type PTs active on pharmaceutically relevant scaffolds.

## Experimental Section

### General Experimental Procedures

The ^1^H NMR of the enriched fraction from the *Ri* DMATS expression strain was recorded on a Bruker Avance NEO 600 MHz spectrometer equipped with a Bruker 5 mm CryoProbe Prodigy BBO at the NMR facility of the Stratingh Institute for Chemistry, University of Groningen. The ^1^H and ^13^C NMR spectra for reference compounds were collected on an Agilent 400 MHz spectrometer equipped with a Varian 5 mm OneNMR probe at the NMR facility of the Stratingh Institute for Chemistry, University of Groningen. Chemical shifts are reported in delta (δ) units using residual protio-solvent as internal standard (CH_3_OH δ 3.35).

HRMS and MS/MS spectra of reference compounds were recorded in positive mode on a Thermo Scientific Q Exactive plus hybrid quadrupole-orbitrap mass spectrometer (Thermo Fisher Scientific) at a resolution of 70,000, following direct infusion. Flow rate was set at 100 μL/min and scan range was set at 150–600 *m*/*z*. The spectra were inspected and processed using the software MZmine 3.^47^

PCR reactions were carried out using 2x Q5 PCR master mix (New England Biolabs) and 1 μL of template (∼10 ng genomic DNA) in a total volume of 25 μL, according to manufacturer’s instructions. Primers and other gene-specific parameters are listed in Table S3. Standard procedures were used for cloning the assembled constructs in competent *E. coli* DH5α cells. Selection of positive clones was carried using LB agar plates supplemented with ampicillin 100 μg/mL.

N-Fmoc-(l)-tyrosine and N-Acetyl-l-tyrosine ethyl ester monohydrate used for the synthesis of reference compounds were purchased from MilliporeSigma.

### Construction of Expression Vectors

The integrative expression vectors pTYargB-eGFPac and pTYadeA-eGFPac were kindly provided by Dr. Colin Lazarus from the University of Bristol, UK. These were digested with FastDigest NotI and PacI and dephosphorylated with FastAP alkaline phosphatase (Thermo Fisher Scientific). The ready-to-use vector fragments were subsequently separated from the eGFPac inserts by gel purification using the QIAquick Gel Extraction Kit (Qiagen). The genes encoding *L. lupina* [GenBank acc. no. KAF6225851.1], and *L. columbiana* [GenBank acc. no. KAF6239039.1] DMATS were amplified from the genomic DNA of wild isolates collected in Spokane County, Washington, USA, for previous studies.^20^ Preliminary sequencing results showed a frame shift in the putative ORF of *L. columbiana* DMATS. This revealed that the actual, shorter, ORF was almost identical to that of *L. lupina* DMATS (Table S4), thus the same primers were used to amplify both genes (Table S3). *As* DMATS was amplified from genomic DNA extracted from mycelium of *A. strigata* CBS 123363 (isolated from limestone in the San Jacinto Mts., San Bernadino County, CA, USA), obtained from the Westerdijk Institute strain collection (Utrecht, The Netherlands). The three genes were cloned by sticky-end ligation in the inducible *amyB* cassette of the pTYargB vector. The gene encoding *Ri* DMATS was synthesized by Twist Bioscience without codon optimization, and further amplified via PCR to add 30bp overlaps for Gibson cloning.^48^ The isothermal method (1 h incubation at 50 °C) was used to assemble the insert in the *amyB* cassette of the pTYadeA vector. Direct-colony PCR was used to pick positive transformants, and the corresponding plasmids extracted using QIAprep Spin Miniprep Kit (Qiagen) were sent to Macrogen Europe for verification by Sanger sequencing.

### Genetic Transformation of *A. oryzae* NSAR1

*A. oryzae* NSAR1 (ΔargB, adeA-, sC-, and niaD-)^24^ was kindly provided by Prof. Jun-ichi Maruyama from the University of Tokyo, Japan. The fungus was cultivated on DPY agar plates for routine passages (20 g/L glucose; 10 g/L peptone; 5 g/L yeast extract; 0.5 g/L MgSO_4_ · 7H_2_O; 5 g/L KH_2_PO_4_; microagar 15 g/L in ddH_2_O; pH 5.5). To achieve profuse sporulation for long-term storage and inoculation of expression cultures, NSAR1 and transformant strains were cultivated on DPY-KCl agar plates (5 g/L glucose; 10 g/L peptone; 5 g/L yeast extract; 0.5 g/L MgSO_4_ · 7H_2_O; 5 g/L KH_2_PO_4_; 45 g/L KCl; 1 mL/L Hutner’s trace element solution;^49^ microagar 15 g/L in ddH_2_O; pH 5.5). Spores were harvested by flooding 7-day old colonies with 5 mL of spore harvest solution (8.5 g/L NaCl; 1 mL/L Tween 80 in ddH_2_O) and manual scraping with sterile loops. Spore solutions were kept at 4 °C for short-term storage or mixed with 45% glycerol 1:2, snap frozen in liquid N_2_, and kept at −80 °C for long-term storage. Protoplasts of *A. oryzae* were obtained as previously described for other *Aspergillus* species,^50^ with some modifications. Briefly, approximately 1 × 10^8^ conidiospores were inoculated in 25 mL liquid DPY medium and grown overnight (∼18h) at 30 °C, 150 rpm. The following day, mycelium was collected by filtration on sterile Miracloth (MilliporeSigma), washed with 5–10 mL of fresh DPY medium, and transferred to a clean 50 mL tube. Eight mL of DPY were then added to the mycelium, which was coarsely disrupted with a pipet tip. To this, eight mL of 2x protoplasting solution (1.28 g of VinoTaste Pro (Novozymes) in 10 mL KC buffer = 82 g/L KCl; 21 g/L citric acid monohydrate in ddH_2_O; pH 5.8) were added. The total content of the tube was transferred into a sterile 125 mL cultivation flask and incubated at 30 °C, 100 rpm. Protoplast formation was monitored every 30 min by examining 10 μL samples under an optical microscope, until a sufficient level was achieved (1.5–2 h). At this point, 16 mL of 0.6 M KCl were added to the flask, and the mixture was pipetted up and down to release more protoplasts, and filtered again through Miracloth to remove undigested hyphae. The filtrate was centrifuged at 1800g, 10 min, to collect the protoplasts. The supernatant was discarded, and the pellet washed with 2 mL 0.6 KCl and centrifuged at 2400g, 3 min. This procedure was repeated twice. Finally, the pellet was resuspended in 1 mL KTC buffer (45 g/L KCl; 5.5 g/L CaCl_2_; 10 mM Tris-HCl in ddH_2_O; pH 7.5) and centrifuged at 2400g, 3 min, one last time. The protoplasts were then resuspended in 600–800 μL KTC buffer, and kept on ice for at least 1 h before proceeding with the following steps. For each transformation, 100 μL of protoplasts were mixed with 1–2 μg (max 10 μL) of the pTY expression vector bearing the lichen DMATS gene (or control eGFP) and 50 μL of PEG-KTC buffer (KTC + 25% w/v PEG4000), and incubated at room temperature for 25 min. After this step, 1 mL of PEG-KTC buffer was added to the mixture, which was incubated on ice for an additional 25 min. Finally, 4 mL of KTC were added, and the transformation mix was centrifuged at 2400g, 3 min. The supernatant was discarded, and the pellet resuspended in 200 μL KTC. These were plated (2 × 100 μL) on 2 TMM agar plates (342.3 g/L sucrose; 2 g/L NH_4_Cl; 1 g/L (NH_4_)_2_SO_4_; 0.5 g/L NaCl; 0.5 g/L KCl; MgSO_4_ · 7H_2_O; 1 g/L KH_2_PO_4_; 1 mL/L Hutner’s trace element solution; methionine 1.5 g/L: 1 g/L arginine; 0.1 g/L adenine; microagar 15 g/L in ddH_2_O; pH 5.5). Arginine or adenine were omitted to select for the vectors bearing the corresponding auxotrophic marker. The protoplasts were regenerated at 30 °C for 5 days, when individual colonies were picked and transferred to new DPY-KCl plates for sporulation and purification of genetically pure clones. For each strain, 4 individual clones were selected and used for the following experiments.

### Cultivation and Metabolite Extraction

The DMATS and eGFP (control) expression strains were inoculated with sterile cotton sticks from their respective spore suspensions onto MPY agar plates (30 g/L maltose; 10 g/L peptone; 5 g/L yeast extract; 0.5 g/L MgSO_4_ · 7H_2_O; 5 g/L KH_2_PO_4_; microagar 15 g/L in ddH_2_O; pH 5.5), where maltose was used as inducer for the *amyB* cassette.^51^ The plates were incubated in the dark at 30 °C for 5 days and the control strains checked over a transilluminator on day 3 to confirm eGFP expression and, thus, successful induction. For extraction of SMs, the whole agar pads (agar and mycelium) were cut into pieces of roughly 1 cm^3^ and transferred to 50 mL polypropylene tubes, then extracted once with 25 mL of 9:1 EtOAc–MeOH (v/v) supplemented with 0.1% formic acid, while being sonicated in a sonication bath for 1 h. Prior to extraction, all samples were spiked with 10 μL of caffeine standard solution (10 mg/mL) to validate the extraction procedure. The organic extracts were collected in clean glass vials and dried under a gentle stream of N_2_ at room temperature. The dry residues were resuspended in 1 mL of 1:1 MeOH-ultrapure H_2_O (v/v) supplemented with 0.1% formic acid by pipetting and vortexing, filtered with 0.45 μm PTFE filters, and stored at −20 °C until further analysis.

### HPLC-MS-DAD Analysis of Fungal Extracts

HPLC-MS-DAD analysis of the fungal extract was first carried out using a low-resolution Waters Acquity Arc HPLC system coupled to a 2998 PDA detector and a QDa single-quadrupole mass detector. A Waters XBridge BEH C18 reversed-phase column was applied for separation (50 mm × 2.1 mm I.D., 3.5 μm, 130 Å particles) which was maintained at 40 °C. The mobile phase consisted of a gradient of solution A (0.1% formic acid in ultrapure H_2_O) and solution B (0.1% formic acid in CH_3_CN). A split gradient was used: 0–2 min 5% B, 2–10 min linear increase to 50% B, 10–15 min linear increase to 90% B, 15–17 min held at 90% B, 17–17.01 min decrease to 5% B, and 17.01–20 min held at 5% B. The injection volume was 2 μL, and the flow rate was set to 0.5 mL/min. MS analysis was carried out in positive mode, with the following parameters: probe temperature of 600 °C; capillary voltage of 1.0 kV; cone voltage of 15 V; scan range 100–1250 *m*/*z*. For diode array detection, the wavelength range was set at 190–800 nm. Data obtained via these experiments was analyzed using the proprietary software MassLynx.

HRMS and MS/MS analyses of fungal extracts were carried out using a Shimadzu Nexera X2 high performance liquid chromatography (HPLC) system with a binary LC20ADXR pump coupled to a Thermo Scientific Q Exactive plus hybrid quadrupole-orbitrap mass spectrometer. A Kinetex EVO C18 reversed-phase column was applied for HPLC separations (100 mm × 2.1 mm I.D., 2.6 μm, 100 Å particles) (Phenomenex), which was maintained at 50 °C. The mobile phase consisted of a gradient of solution A (0.1% formic acid in ultrapure H_2_O) and solution B (0.1% formic acid in CH_3_CN). A linear gradient was used: 0–3 min 5% B, 3–51 min linear increase to 90% B, 51–55 min held at 90% B, 55–55.01 min decrease to 5% B, and 55.01–60 min held at 5% B. The injection volume was 2 μL, and the flow rate was set to 0.25 mL/min. MS and MS/MS analyses were performed with electrospray ionization (ESI) in positive mode at a spray voltage of 3.5 kV, and sheath and auxiliary gas flow set at 60 and 11, respectively. The ion transfer tube temperature was 300 °C. Spectra were acquired in data-dependent mode with a survey scan at *m*/*z* 100–1500 at a resolution of 70,000, followed by MS/MS fragmentation of the top 5 precursor ions at a resolution of 17,500. A normalized collision energy of 30 was used for fragmentation, and fragmented precursor ions were dynamically excluded for 10 s. Data obtained by these experiments was analyzed using MZmine 3.^47^

### Large Scale Cultivation and Semipreparative HPLC

For large scale cultivation and purification of fungal extracts, we inoculated the *Ri* DMATS expression strain on eight large 125 mL MPY agar plates (total volume 1 L). The spores were transferred with a sterile cotton stick as before, but this time on 4 equidistant spots on each agar plate. The cultures were then incubated in the dark at 30 °C for 7 days, enclosed in a box to prevent excessive evaporation. For extraction of SMs, the whole agar pads (agar and mycelium) were cut into pieces of roughly 1 cm^3^ and transferred in a clean borosilicate glass bottle. The total amount of biomass and agar (approximately 1 L) was extracted twice with equal amounts of 9:1 EtOAc–MeOH (v/v) supplemented with 0.1% formic acid. The extraction mixture was sonicated in a sonication bath for 1 h each time. The combined organic extract was collected in a clean borosilicate glass bottle with the help of a separatory funnel. The extract was then concentrated under reduced pressure with a rotary evaporator to yield ∼20 mL of concentrated extract. This was transferred to a clean glass vial and dried under a gentle stream of N_2_ until complete evaporation of the solvents, yielding 989 mg of extract. The residue was resuspended in 9.89 mL of 1:1 MeOH-ultrapure H_2_O (v/v) supplemented with 0.1% formic acid to achieve a final concentration of ∼100 μg/μL, sonicated for 1 h, and filtered with 0.45 μm PTFE filters to remove any undissolved material.

For purification, high-performance liquid chromatography was carried out on a HPLC Shimadzu system, equipped with a dual LC-20AD pump and an SPD-20M20A photodiode array detector. A Macherey-Nagel VarioPrep NucleoDur C18 ec reversed-phase column was applied for separation (250 × 10 mm I.D., 5 μm, 110 Å particles) which was maintained at room temperature (∼25 °C). The mobile phase consisted of a gradient of solution A (ultrapure H_2_O) and solution B (CH_3_CN). Following method development, the following linear gradient was established: 0–15 min 5% B, 15–35 min linear increase to 60% B, 35–40 min held at 60% B, 40–40.01 min decrease to 5% B, and 40.01–60 min held at 5% B. The injection volume was 50 μL, and the flow rate was set to 5 mL/min. Based on comparison between total absorbance chromatograms and total ion chromatograms generated previously, 7 fractions were collected in a scout run, and analyzed on the Waters HPLC-MS-DAD system described above. The mobile phase consisted of a gradient of solution A (0.1% formic acid in ultrapure H_2_O) and solution B (0.1% formic acid in CH_3_CN). A shorter linear gradient was used: 0–2 min 5% B, 2–5 min linear increase to 90% B, 5–7 min held at 90% B, 7–7.01 min decrease to 5% B, and 7.01–10 min held at 5% B. The injection volume was 2 μL, and the flow rate was set to 0.5 mL/min. Fraction 6 (F6, t_R_ 28.1–28.4 min, Figure 2a) was determined to be the fraction containing both compounds **1** and **2**. Repeat injections were performed until a total volume of ∼40 mL of F6 was collected. The combined fractions were concentrated under reduced pressure to yield an aqueous residue of about 15 mL, which was then snap-frozen in liquid N_2_ and lyophilized overnight to obtain 2.1 mg dry residue (off-white powder). This was dissolved in 300 μL of CD_3_OD, transferred to a Norell Select Series 3 mm tube and analyzed via ^1^H NMR (Figure S2 and Figure 2b).

### Chemical Synthesis of Reference Compounds

#### Diprenyl-(l)-tyrosine

To a suspension of N-Fmoc-(l)-tyrosine (360 mg, 892 μmol) and K_2_CO_3_ (394 mg, 3.2 equiv) in CH_3_CN (1.8 mL), was added prenyl bromide (247 μL, 2.4 equiv). The reaction was stirred for 18 h at rt (Scheme S1). Solids were filtered and washed with EtOAc (3 × 5 mL). The combined filtrates were evaporated to dryness. The crude residue was purified by flash chromatography. Fractions containing spots of Rf = 0.23 in 30% EtOAc in pentanes were combined and evaporated. The reaction afforded 55 mg of the desired product (19%).

#### 4-*O*-Prenyl-(l)-tyrosine

Diprenyl-(l)-tyrosine (55 mg, 173 μmol) was suspended in water (0.8 mL), and LiOH·H_2_O (4.1 mg, 1.0 equiv) was added. THF (0.8 mL) was added, and the resulting milky mixture was stirred overnight (Scheme S2). TLC showed complete conversion. The volatiles were evaporated, and the residue was azeotrope with toluene (5 × 5 mL) until solid. The solid was triturated with MTBE (2 × 5 mL) and pentane (5 mL). Reaction afforded 21 mg (48%) of white solid.

^1^H NMR (400 MHz, CD_3_OD) δ 7.21–7.12 (m, 2H), 6.87–6.79 (m, 2H), 5.44 (tdd, J = 6.8, 2.9, 1.5 Hz, 1H), 4.49 (d, J = 6.6 Hz, 2H), 3.45 (dd, J = 8.2, 4.6 Hz, 1H), 3.06 (dd, J = 13.7, 4.6 Hz, 1H), 2.74 (dd, J = 13.7, 8.1 Hz, 1H), 1.77 (s, 3H), 1.74 (s, 3H).

^13^C NMR (101 MHz, CD_3_OD) δ 159.03, 138.34, 131.63, 131.40, 121.42, 115.79, 65.80, 58.85, 41.39, 25.84, 18.16.

HRESIMS *m*/*z* 250.1437 [M + H]^+^ (calcd for C_14_H_20_NO_3_^+^, 250.1443).

#### 4-*O*-Prenyl-N-acetyl-(l)-tyrosine Ethyl Ester

N-acetyl-(l)-tyrosine ethyl ester monohydrate (125.6 mg, 500 μmol) was suspended in CH_3_CN (1.0 mL). K_2_CO_3_ (172.7 mg, 2.5 equiv) followed by prenyl bromide (69.2 μL, 1.2 equiv) were added and the resulting suspension was stirred for 2 days at rt (Scheme S3). The reaction mixture was diluted with EtOAc (20 mL), washed with water (2 × 10 mL) and brine (10 mL), filtered over a phase separator, and evaporated to dryness. The reaction afforded 158.0 mg of a viscous liquid that was used without further manipulation.

#### 4-*O*-Prenyl-N-acetyl-(l)-tyrosine

4-O-prenyl-N-acetyl-(l)-tyrosine ethyl ester (159.7 mg, 500 μmol) was suspended in water (2.5 mL), and LiOH·H_2_O (21.0 mg, 1.0 equiv) was added. The reaction was stirred overnight (Scheme S4). After TLC showed the complete conversion, the volume was reduced to ca. 20% by rotavap. The residue was diluted with water (5 mL), transferred into a separatory funnel and extracted with MTBE (3 × 10 mL). The extracts were discarded. The aqueous phase was acidified to pH = 2 by careful addition of HCl (ca. 1 M, ca. 800 μL). The aqueous phase was extracted with EtOAc (3 × 10 mL). Combined extracts were washed with brine, filtered over a phase separator and evaporated to dryness. Reaction afforded 140 mg (76%) of white waxy solid. ^1^H NMR suggests an EtOAc solvate (ca. 1/1 desired product/EtOAc).

^1^H NMR (400 MHz, CD_3_OD) δ 7.12 (d, *J* = 8.6 Hz, 2H), 6.82 (d, *J* = 8.6 Hz, 2H), 5.44 (ddt, *J* = 6.6, 5.2, 1.4 Hz, 1H), 4.60 (dd, *J* = 8.8, 5.1 Hz, 1H), 4.49 (d, *J* = 6.4 Hz, 2H), 4.10 (q, *J* = 7.1 Hz, 2H, EtOAc), 3.12 (dd, *J* = 14.0, 5.1 Hz, 1H), 2.87 (dd, *J* = 14.0, 8.9 Hz, 1H), 2.01 (s, 3H, EtOAc), 1.99 (s, 1H), 1.91 (s, 3H), 1.78 (s, 3H), 1.73 (d, *J* = 1.3 Hz, 3H), 1.24 (t, *J* = 7.1 Hz, 3H, EtOAc).

^13^C NMR (101 MHz, cd_3_od) δ 174.90, 173.10, 172.98 (EtOAc), 159.21, 138.43, 131.18, 130.37, 121.33, 115.69, 65.81, 61.53 (EtOAc), 55.38, 37.66, 25.84, 22.31, 20.85 (EtOAc), 18.16, 14.46 (EtOAc).

HRESIMS *m*/*z* 292.1541 [M + H]^+^ (calcd for C_16_H_22_NO_4_^+^ 292.1549).

### Sourcing Putative Lichen DMATS Sequences

Annotated genomes of 21 lichen-forming fungi were kindly provided by Dr. Wonyong Kim from the Korean Lichen Research Institute, Sunchon National University, Suncheon, South Korea.^14^ The remaining 17 were retrieved from the NCBI and JGI Mycocosm databases,^52,53^ accessed in September, 2021. The annotated genomes were used as input for biosynthetic gene cluster prediction by antiSMASH v7.0.0^22^ (parameters: --taxon fungi --cassis --clusterhmmer --genefinding-tool none). Of the 2038 clusters identified, 60 were predicted to contain a DMATS-type prenyltransferase. From there, 61 putative lichen DMATS were retrieved.

### Sequence Similarity Network Analysis

All protein sequences that are part of the fungal aromatic prenyltransferase DMATS-type family (IPR012148) were retrieved from the InterPro database^26^ and combined with the DMATS sequences mined manually from lichen genomes, yielding a total of 1446 unique sequences. In total, 67 sequences originate from lichen organisms: 6 were already present in the InterPro data set and 61 were added based on our manual search, described above. The resulting multi-FASTA file was used as input for generating a sequence similarity network with the Enzyme Similarity Tool of the Enzyme Function Initiative (EFI-EST);^27^ edge selection cutoff: alignment score >82 (corresponding to sequence ID > 34.23%). The network was colored using the Color SSN utility and visualized in Cytoscape v3.8.2^54^ with the yFiles organic layout.

### Multiple Sequence Alignment Analysis

The MSA analysis of biochemically characterized fungal DMATS-type PTs and target lichen PTs (and *Uf* DMATS) was performed using MEGA 11,^55^ using the MUSCLE algorithm^56^ with default settings. The resulting alignment was exported in FASTA format and used as input for the phylogenetic analysis. The software IQ-TREE v2.2.2.6^57^ was used to build a consensus phylogenetic tree with the bootstrap method using the following parameters: --seqtype AA -m TEST -b 1000 (number of replicates = 1000). The final tree was visualized and refined with the online tool Interactive Tree Of Life (iTOL) v6.^58^

### Modeling and Molecular Docking

AlphaFold2 Colab was utilized to model the structures of *Ri* DMATS and *As* DMATS based on their amino acid sequences,^42,43^ followed by an additional relaxation step employing Amber^59^ to ensure precise positioning of the residue side chains. Docking of DMAPP and l-tyrosine into the active site of the generated *Ri* DMATS model was carried out using AutoDock Vina.^60,61^ Following this, a 20 ns molecular dynamics (MD) simulation was performed using YASARA v23.5.19 (YASARA Biosciences). For that, the YASARA dynamics software package incorporating the YAMBER3 force field was used.^62^ Initially, the enzyme–substrate complexes were cleaned, minimized, and placed within a rectangular simulation cell, with the distances between the protein and the periodic boundaries of the cell maintained at a minimum of 7.5 Å. The Berendsen thermostat algorithm^63^ and manometer algorithm were used to maintain consistent temperature and pressure values during MD simulations. The physiological conditions of the simulation cells were set to mimic 298 K, pH 7.4, and 0.9% NaCl. Temperature was gradually increased from 5 to 298 K over 30 ps, followed by a 4970 ps equilibration period before the production phase simulation of 20,000 ps. Snapshots were captured every 25 ps. Finally, snapshots of the DMAPP and l-Tyr–bound *Ri* DMATS model were captured at the end of the simulation to analyze the active site architecture and binding of the substrates.

### Visualization and Other Bioinformatic Tools

For visualization of molecular models and docking, UCSF Chimera v1.17.3 was used.^64^ Custom settings were used to display ribbon and atoms in cartoon style, and flat lighting was applied. Variations of the Wong and Tol colorblind-accessible palettes were used. Clinker^65^ was used, with standard settings, to display and annotate the BGC containing the putative prototype DMATS from *Usnea florida* shown in Figure S13. Jalview^66^ was used to visualize and generate the MSA alignment shown (partially) in Figure S14. CLUSTAL coloring was applied to the amino acid sequence.

## Supporting Information Available

Supplementary figures and tables, synthesis schemes, and NMR spectra of reference compounds (PDF)
Full MSA of biochemically characterized fungal DMATS-type PTs (+ target lichen PTs) (TXT)
SSN file (Cytoscape session) (ZIP)
Raw NMR data for reference compound PFO163 (compound **1**) (ZIP)
Raw NMR data for reference compound PFO173 (compound **2**) (ZIP)
