# Supplementary material for: Discovery and Heterologous Expression of Functional 4-O-Dimethylallyl-l-tyrosine Synthases from Lichen-Forming Fungi
Source: J Nat Prod. 2024 Sep 10;87(9):2243–54. doi: 10.1021/acs.jnatprod.4c00619 (PMC11443524; doi:10.1021/acs.jnatprod.4c00619)
Supplement: Supplementary file 1 — np4c00619_si_001.pdf [file np4c00619_si_001.pdf]

Supporting information for

Discovery and heterologous expression of functional 4-O-dimethylallyl-L-tyrosine synthases from lichen-forming fungi

Riccardo Iacovelli[1†], Siqi He[1‡], Nika Sokolova[1‡], Iris Lokhorst[1], Maikel Borg[1], Peter Fodran[1], and Kristina Haslinger[1]*

[1]Department of Chemical and Pharmaceutical Biology, Groningen Research Institute of Pharmacy, University of Groningen, 9713 AV Groningen, The Netherlands

[†]Current address: VTT Technical Research Centre of Finland Ltd, 02150 Espoo, Finland

*To whom correspondence should be addressed

Email: k.haslinger@rug.nl

Telephone: +31 (0)63 192 1108; Fax: +31 (0)50 363 3000

Contents

Supporting schemes 2

Supporting tables 3

Supporting figures 7

References 23

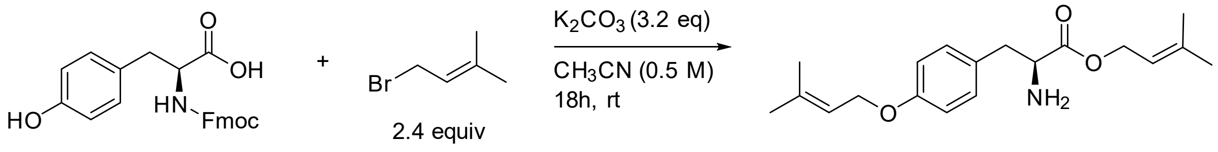

Scheme S1. Synthesis scheme for diprenyl-(L)-tyrosine.

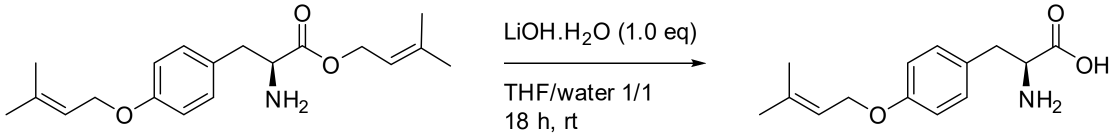

Scheme S2. Synthesis scheme for 4-O-prenyl-(L)-tyrosine

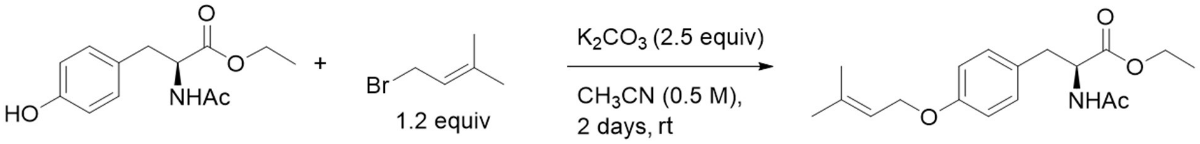

Scheme S3. Synthesis scheme for 4-O-prenyl-N-acetyl-(L)-tyrosine ethyl ester.

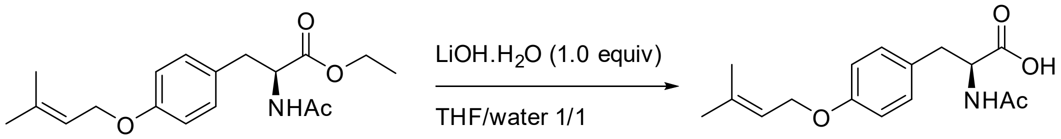

Scheme S4. Synthesis scheme for 4-O-prenyl-N-acetyl-(L)-tyrosine.

Table S1. Genomic location of target DMATS from lichen-forming fungi.

Gene	Organism & genome assembly	Scaffold/contig	region ID	Location BGC and gene (nt)
As DMATS	*Acarospora strigata* CBS 132363[1] – JGI Mycocosm database[2]	scaffold 8982	AS-522.1	43,412 – 87,968 (58,206 – 61,127)
Ri DMATS	*Ramalina intermedia* YAF0013 – unpublished – GenBank acc. no. GCA_003073195.1	scaffold 38 (acc. no. PEKF01000038.1)	RI-146.1	107,028 – 128,413 (117,028 – 118,413)
Ll DMATS (GenBank acc. no. KAF6225851.1	*Letharia lupina* WasteWater1 isolate[3] – GenBank acc. no. GCA_014066315.1	contig 7 (acc. no. JACCJB010000007.1)	LL-28.2	2,058,775 – 2,089,139 (2,077,949 – 2,079,139)
Lc DMATS (GenBank acc. no. KAF6239039.1)	*Letharia columbiana* WasteWater2 isolate[3] – GenBank acc. no. GCA_014066305.1	contig 7 (acc. no. JACCJC010000007.1)	LC-7.2	767,644 – 791,438 (777,644 – 781,438)

Table S2. Comparison of HRMS and MS2 data of compound 1 and 2 (from total fungal extract) with synthetic standards. MS2 spectra were recorded in DDA mode (top 5 peaks per MS1 scan).

Compound	Molecular formula	$[M+H]^+$ precursor (error, ppm)	Top MS/MS fragment ions[a]	
			$[M+H]^+$	Intensity, %[b]
1	$C_{14}H_{19}NO_3$	250.1438 (-2.00)	165.0544	100
			136.0765	52
			69.0698	26
			123.0447	23
			147.0437	19
			119.0491	11
2	$C_{16}H_{22}NO_4$	292.1547 (-0.68)	204.1384	100
			136.0765	100
			246.1495	54
			178.0868	53
			233.1171	47
			182.0816	39
			165.0543	29
			69.0704	20
			250.1456	18
PFO163	$C_{14}H_{19}NO_3$	250.1437 (-2.40)	165.0541	100
			136.0753	46
			69.0701	25
			123.0438	21
			147.0438	17
			119.0495	11
PFO173	$C_{16}H_{22}NO_4$	292.1541 (-2.74)	136.0752	100
			204.1397	84
			178.0862	68
			182.0809	49
			246.1506	41
			233.1184	40
			165.0540	32
			69.0699	28
			250.1422	15
			205.1420	10
			137.0789	10

a. Only fragment ions with % intensity > 10 are shown
b. Peak height relative to main fragment

Table S3. Primers used to clone target lichen DMATS.

Target	FW primer[a] (5' → 3')	RV primer[a] (5' → 3')	T_{ann}	Extension time
Ll DMATS<br>*Lc* DMATS	*ctag*GCGGCCGCatgataggt<br>cgccctctagctctg	*tagc*TTAATTAActatgctttcc<br>atctagcaacatgatagatttc	67 ˚C	30 sec
As DMATS	*atcg*GCGGCCGCatgagttgc<br>ggaggtaacatggac	*atgc*TTAATTAAttaagactgt<br>gacgctttcatgtgc	68 ˚C	30 sec
Ri DMATS	**ctcccttctctgaacaataaacccc**<br>**acagc**gcggccgcatggctggtga<br>ggccagtcaaatg	**cagtaccatcatatactctccaccc**<br>**ttaat**ttatgcataggcctttggagca<br>atataagatgtcaat	72 ˚C	35 sec

a. 4-bp cleavage overhangs are italicized; recognition sites for restriction enzymes NotI (FW) and PacI (RV) are capitalized; 30-bp overlap regions for Gibson cloning of *Ri* DMATS are underlined and in bold.

NB: *L. lupina* and *L. columbiana* are closely related, and the corresponding target DMATS are homologs and display 98.99% amino acid sequence identity.

Table S4. Amino acid sequences of target lichen DMATS.

Enzyme	Length	Sequence
Ll DMATS	396	MIGRPLALLLHEAGYDIHNQYGSLLFFRHCIAGRLGARPTSTGSPQVWKSFM<br>TDDFSPVEYSWCWDTPKGPPRIRFSVDAIGPDAGTQSDPFNQEMTTDLVRHV<br>ESVASNVDWKLFNHFRNAFCEQGLEKRVSEGCDDLEKSHTSSIFMAFELHKS<br>EVAVKAYFVPVKAEQTGRSRLSVLSDSIVSLEKSDLRVGAYDQMLAFMTSDA<br>EGSHLEIVGIAVDCVLPKDSRLKLYVRSPSTSFDSVCAIMTLGGKLNTFPQATL<br>KDFRKLWQLTLGLGEDFAPGANLQAKSHETAGVLYNFDIKAGNLLPEPKVYI<br>PVRHYARNDLAAAEGLASYLKSKGQDRFVESYMRALEGMCTHRFLGSQCG<br>LQTYISCAVQNAQLVLTSYLSPEIYHVARWKA
Lc DMATS	396	MIGRPLALLLHEAGYDIHNQYGSLLFFRHCIAGRLGARPTSTGSPQVWRSFM<br>TDDFSPVEYSWCWDTPKGPPRIRFSVDAIGPDAGTQSDPFNQEMTTDLVRHV<br>ESVASNVDWKLFNHFRNAFCEQGLEKRVSEGCDDLEKSHTSSIFMAFELHKS<br>EVAVKAYFVPVKAEQTGRSRLSVLSDSIVSLEKSDLRVGAYDQMLAFMTSDA<br>EGSHLEIVGIAVDCVLPKDSRLKLYVRSPSTSFDSVCAIMTLGGKLNTFPQAT<br>WKDFRKLWQLTLGLGEDFAPGANLQAKSHETAGVLYNFDIKAGNLLPEPKV<br>YIPVKHYARNDLAAAKGLASYLKSKGQDRFVESYMRALEGMCTHRFLGSQC<br>GLQTYISCAVQNAQLVLTSYLSPEIYHVARWKA
As DMATS	435	MSCGGNMDGLDAGEALNSLSWQNSAGVMLSEMMEMAGYHLQSQRSHLDF<br>FARHVAPALGSHPEIDRKPPPWRSFMTDDGSPIELSWSWSVQEPAPIVRYSIEPI<br>GDRAGLCPDYFNTHTSNELVHIIQRSYQGVDLTGYAHFFKELVVCGEGTFVPK<br>ITKEDGSNSQIFLAFDLLDEKIMLKVYFLPALRARETGQCKLSMVEKAISTLSP<br>HGQSLSGAFSLVCEYIRSLKVGNRPEIEIIAVDCVNPTLSRVKVYLRSRETSFAS<br>VVSMMTLGGRLKELSKEGFATLEELWRLVLSLDPTISTSEPLHLNNRRTAGILY<br>YFELQPSRSYPKPKVYIPVKHYGKSDLKVANGLSSYLKEKGKRLNGMDYRD<br>ALQRLCKHRPLDQGSGLHTYVACAIEDISLAVTAYINPEIYHRPRPANRAPAHR<br>ETSHMKASQS

Ri DMATS	461	MAGEASQMLRSKRDSMSWQHQYERLASARSELLKRVQLGETVTSTPKIWEL<br>ITSLQLSIDEDVRFWWTVLGTPLAILFQKAGYSIESQYQHLLFFYFLVAPELGA<br>RSDGQGLPTTWKSFMTDHFTPIEMSWEWGSNSDGGPTIRYAFEPISAHAGTTL<br>NPLNEGASTRVMHRYSQMIPGCDMTLFHHFAQDLLCYDPSPVSTQGKINSQG<br>HASRCFLAIEFNKSEVMVKAYFFPTFKAIRTNQCPWTMISESILNMPGYSSLM<br>LQSLSSFETFLRCSPEGLNLVPEILAIDCGPPAESRMKIYMRSRSTTFESVRRVM<br>TLDGALRESGLEKGLYELYVLWTLVFWHGRQVAPEASLQSVEHRTAGILYYF<br>NLSQGGQPPSVKVYLPVRHYGYSDCDVAQGVITYLRSRGRSCSTTEYIEALTA<br>IARPKSLGSQRGLQTYLGCSIVGEKLKLTSYIAPKAYA

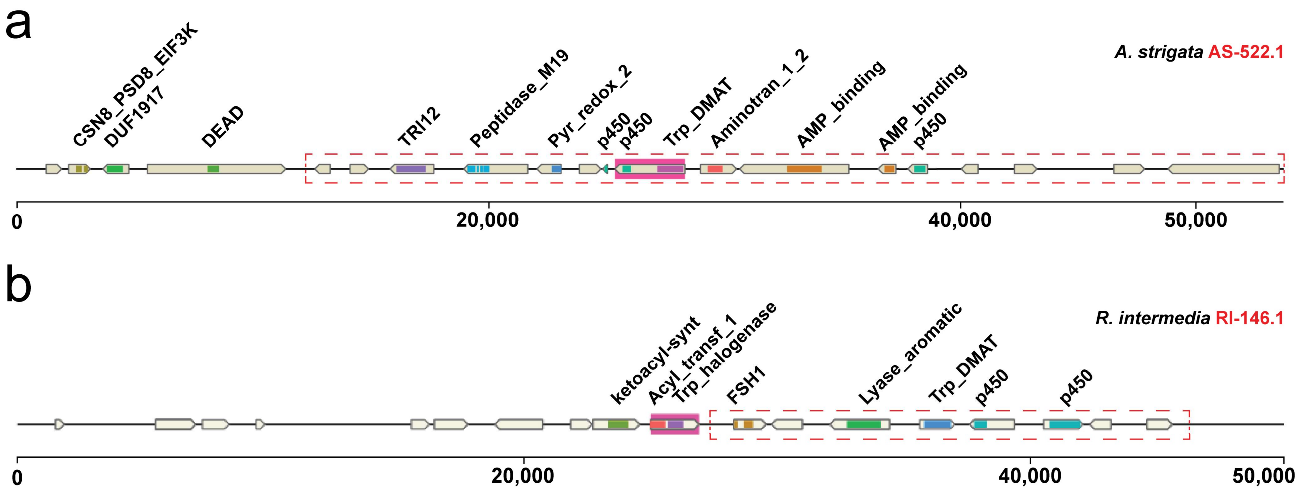

Figure S1. Genome neighborhoods of target DMATS from *A. strigata* and *R. intermedia*. The genomic regions were visualized with the gggenomes R package[4]. The specific PFAM domain hits are highlighted by colors and corresponding labels. Corresponding BGCs as predicted by fungiSMASH[5] are highlighted by red dashed boxes. **(a)** Neighborhood (25kb up- and downstream) of putative di-domain DMATS-P450 from *A. strigata*, highlighted in pink. Sequencing results later revealed that this was, in fact, not a fusion protein. **(b)** Neighborhood (25kb up- and downstream) of putative di-domain acyltransferase-halogenase from *R. intermedia*, highlighted in pink. The DMATS gene subject of this study co-localizes downstream of the di-domain protein and is annotated as Trp_DMAT.

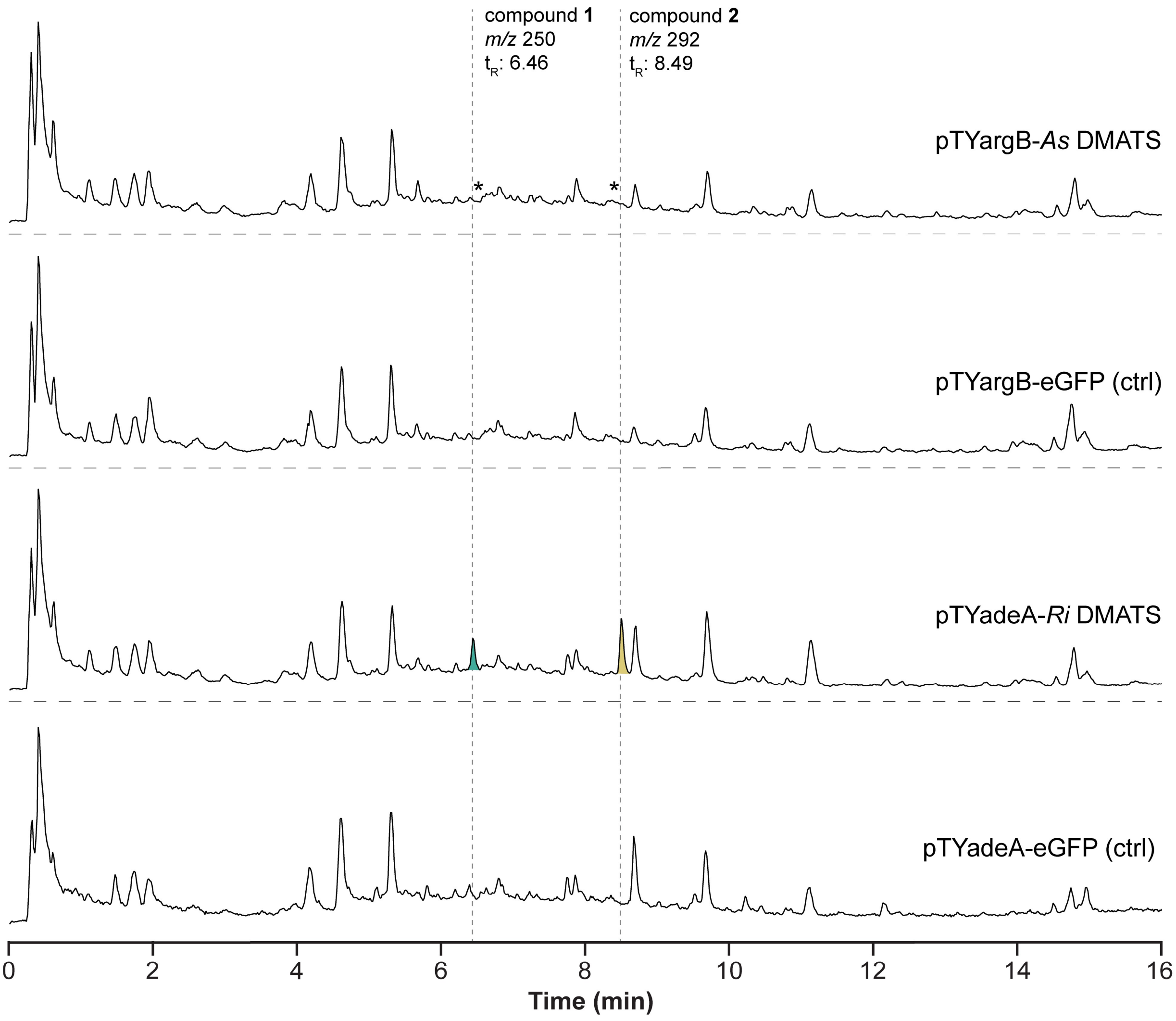

Figure S2. Comparison of Total Ion Chromatograms (TICs) of DMATS expression strains and respective controls. The peaks of compounds **1** and **2** are highlighted in the extract of the *Ri* DMATS expression strain. *The corresponding peaks in the extract of the *As* DMATS expression strain are not visible from the TIC, as their signals fall below background levels. We were only able to detect them upon manual inspection of the extracted ion chromatograms.

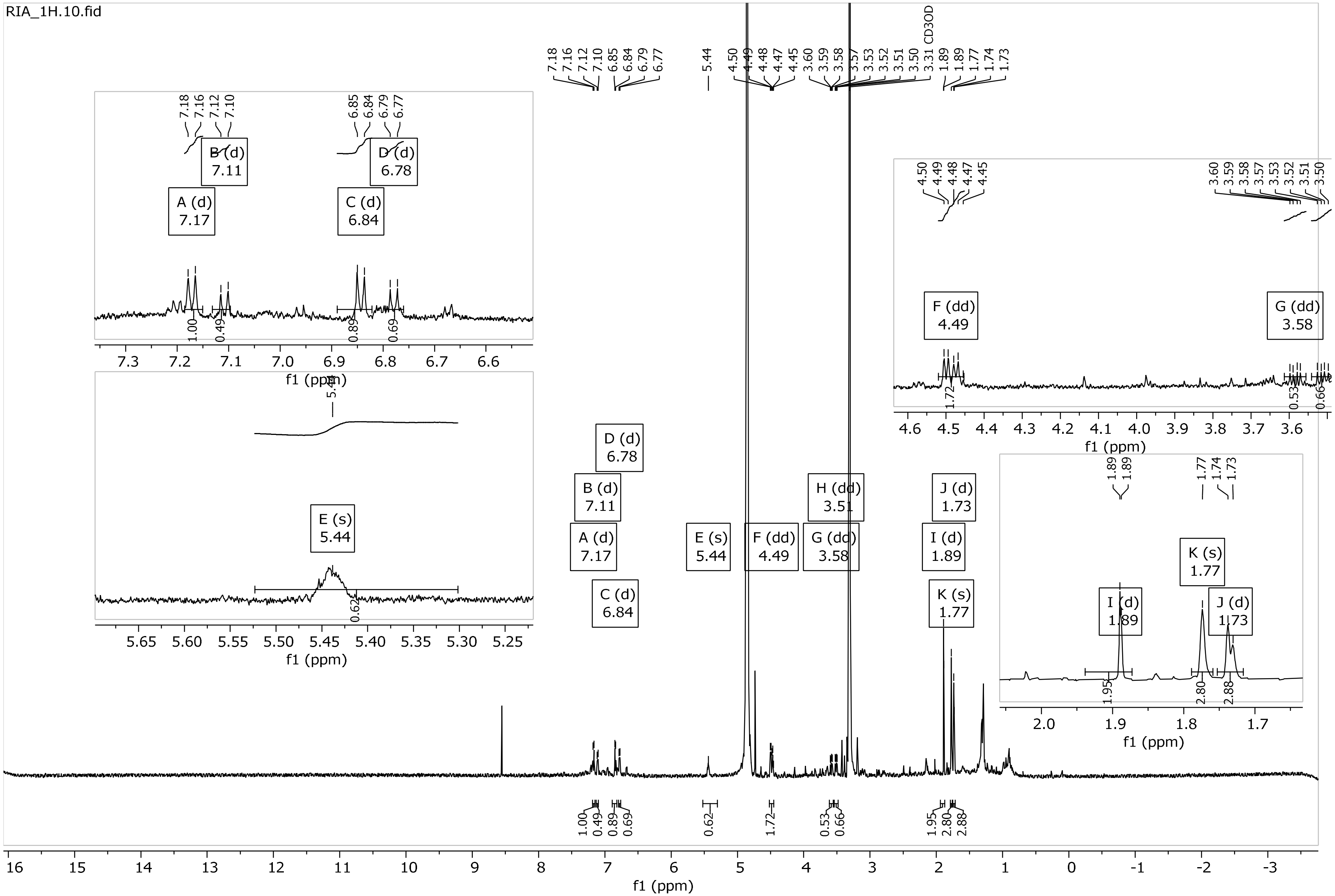

Figure S3. **^{1}H NMR spectrum (600 Mhz) of enriched fraction from *Ri* DMATS extract, containing compound 1 and 2, in CD_3OD.**

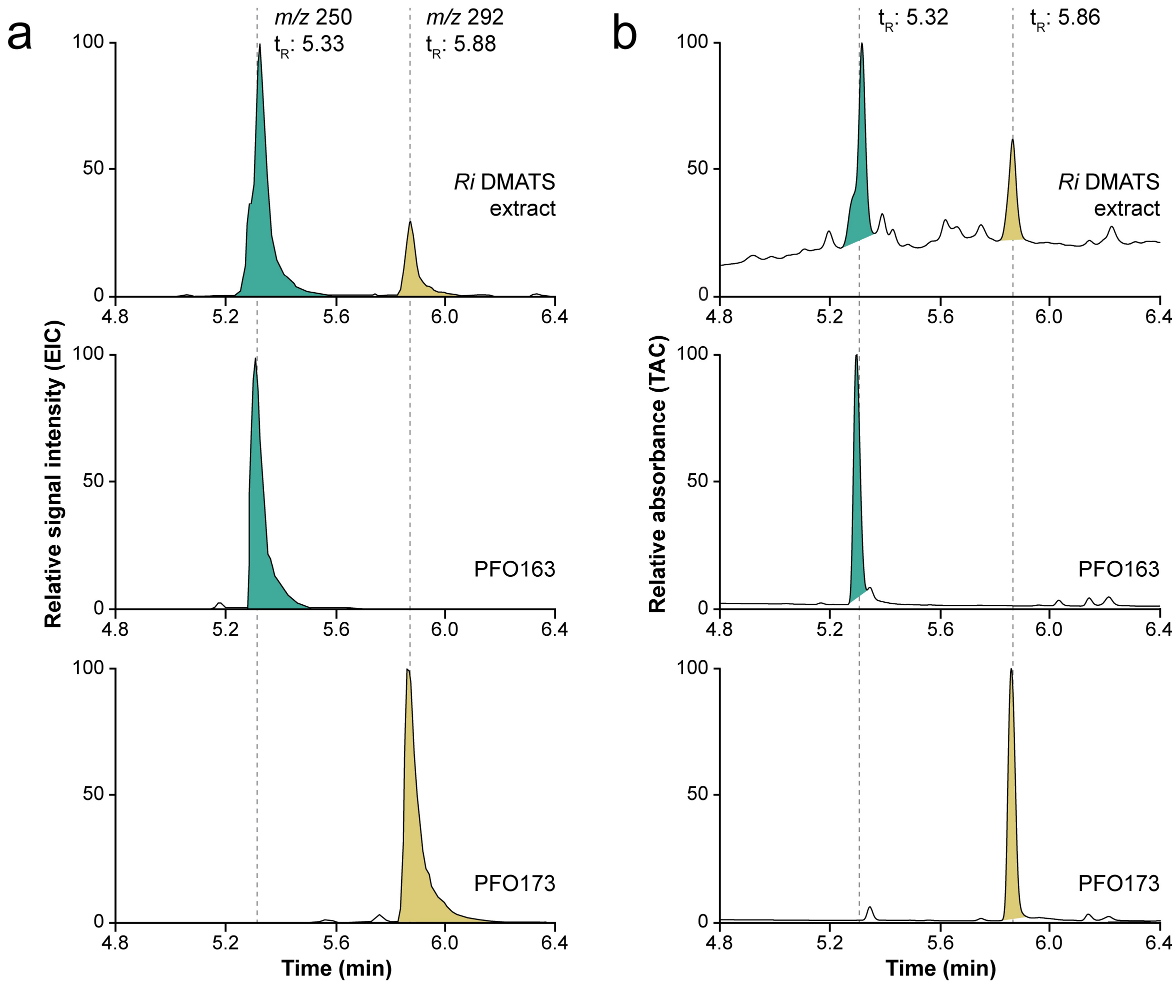

Figure S4. Comparison between chromatograms of the enriched fraction from *Ri* DMATS extract and reference standards PFO163 and PFO173. (a) Extracted ion chromatograms with filters set to *m/z* 250 (± 0.2 Da) and *m/z* 292 (± 0.2 Da) for compound **1** and **2** and their references, respectively, in positive ionization mode (ESI+). **(b)** Total absorbance chromatograms generated by diode array detection, with wavelength range set to 190-800 nm.

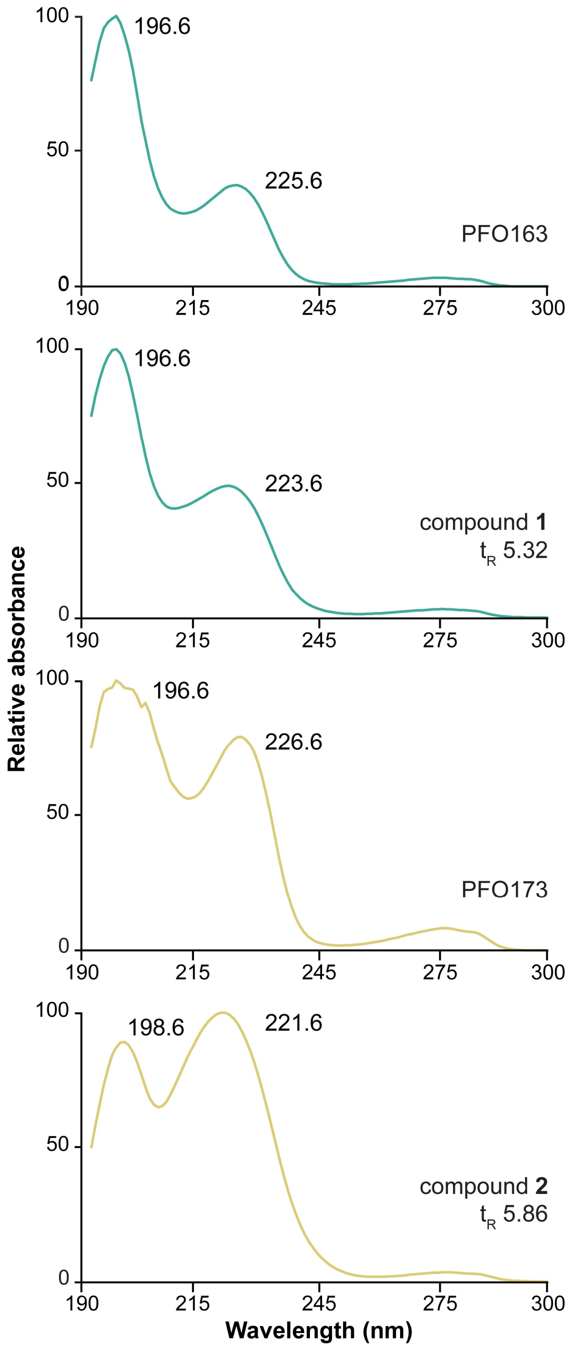

Figure S5. UV spectra of compounds 1 and 2 from enriched fraction of *Ri* DMATS extract compared to reference standards PFO163 and PFO173. Only the 190-300 nm range is shown as the signal over 300 nm fell below baseline levels. The peaks are automatically annotated by the MassLynx software at the highest point of the spectrum.

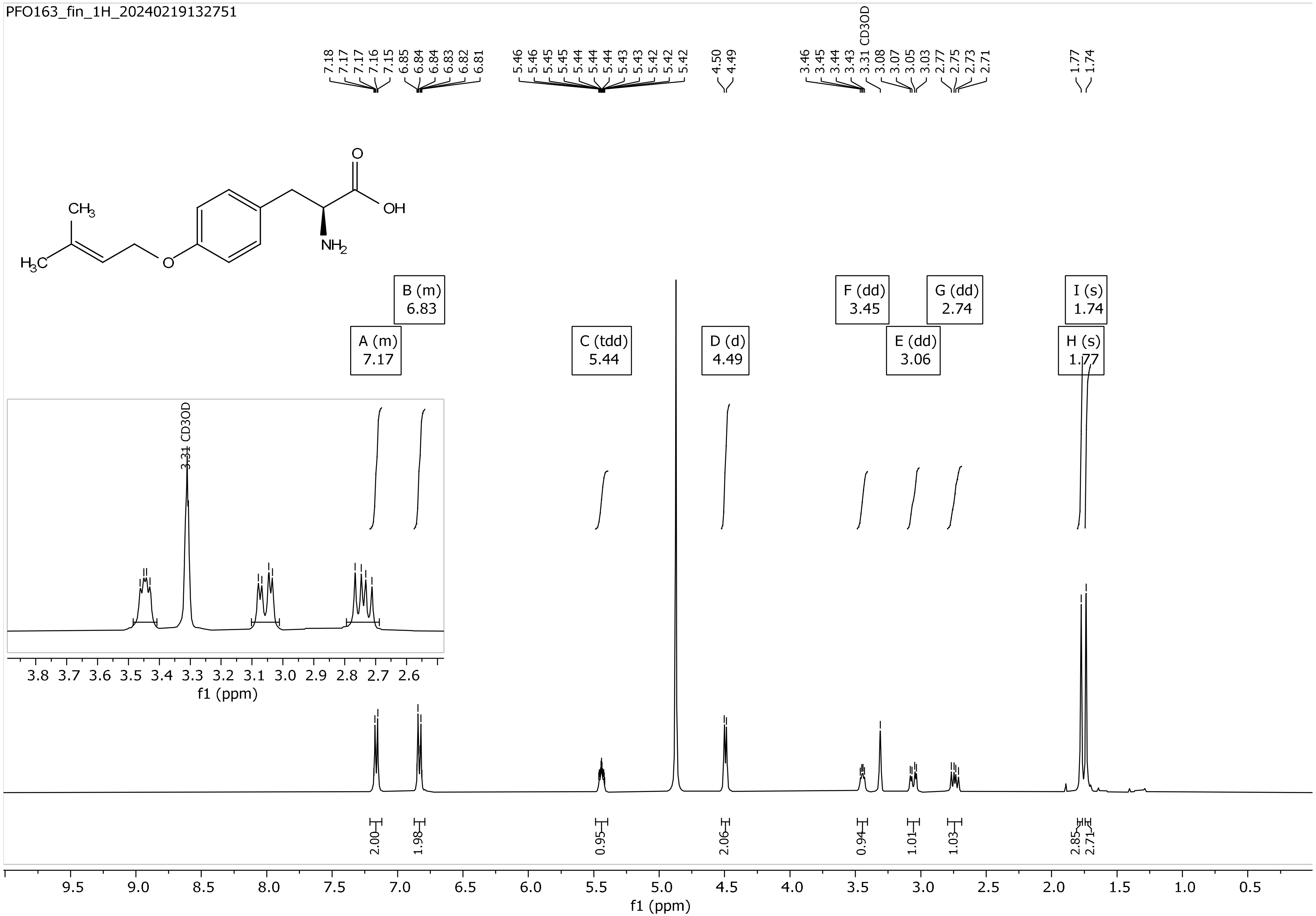

Figure S6. **^{1}H NMR spectrum (400 Mhz) of compound PFO163: 4-O-dimethylallyl-L-tyrosine, in CD_3OD.**

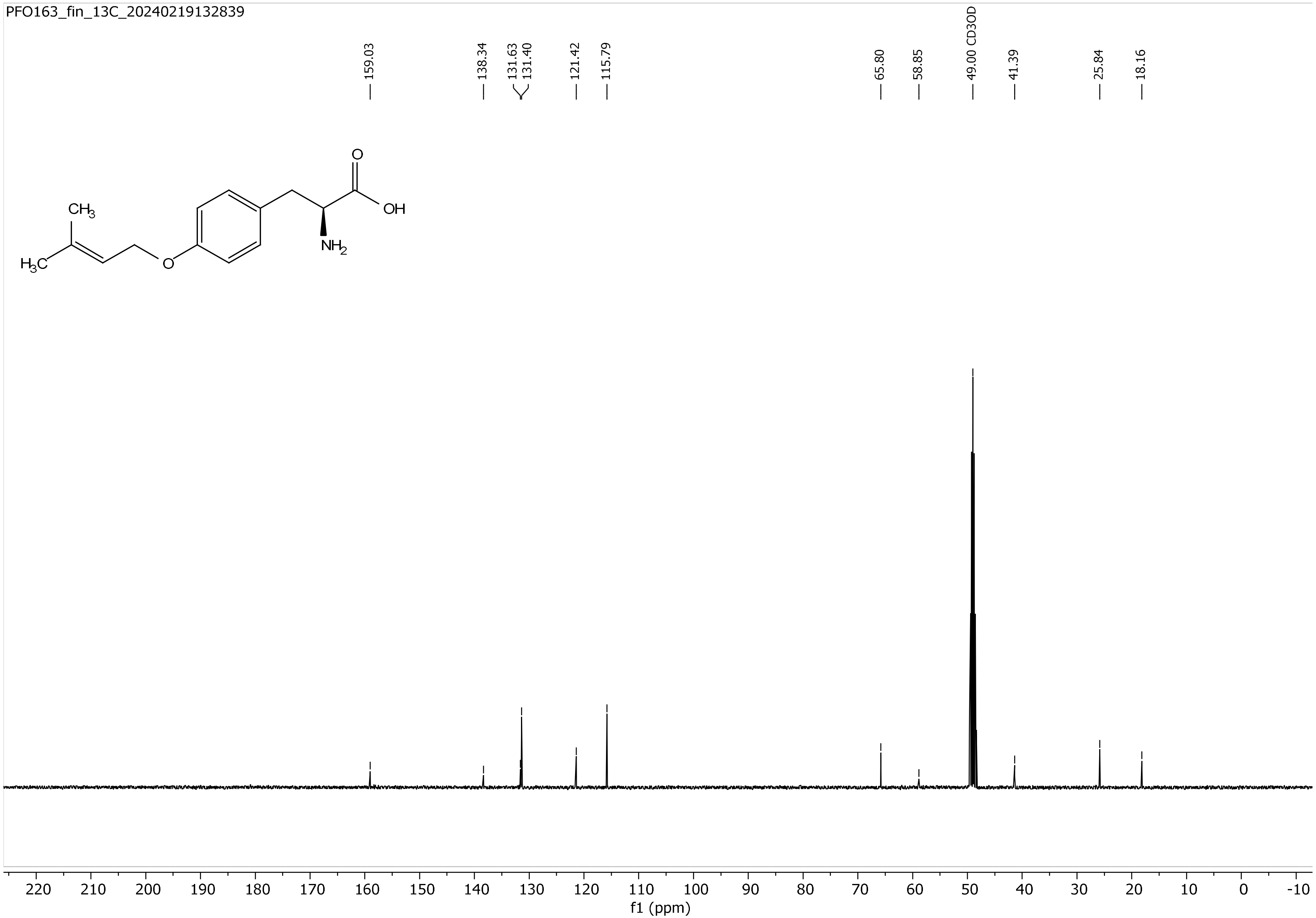

Figure S7. ^{13}C NMR spectrum (101 Mhz) of compound PFO163: 4-O-dimethylallyl-L-tyrosine, in CD_3OD.

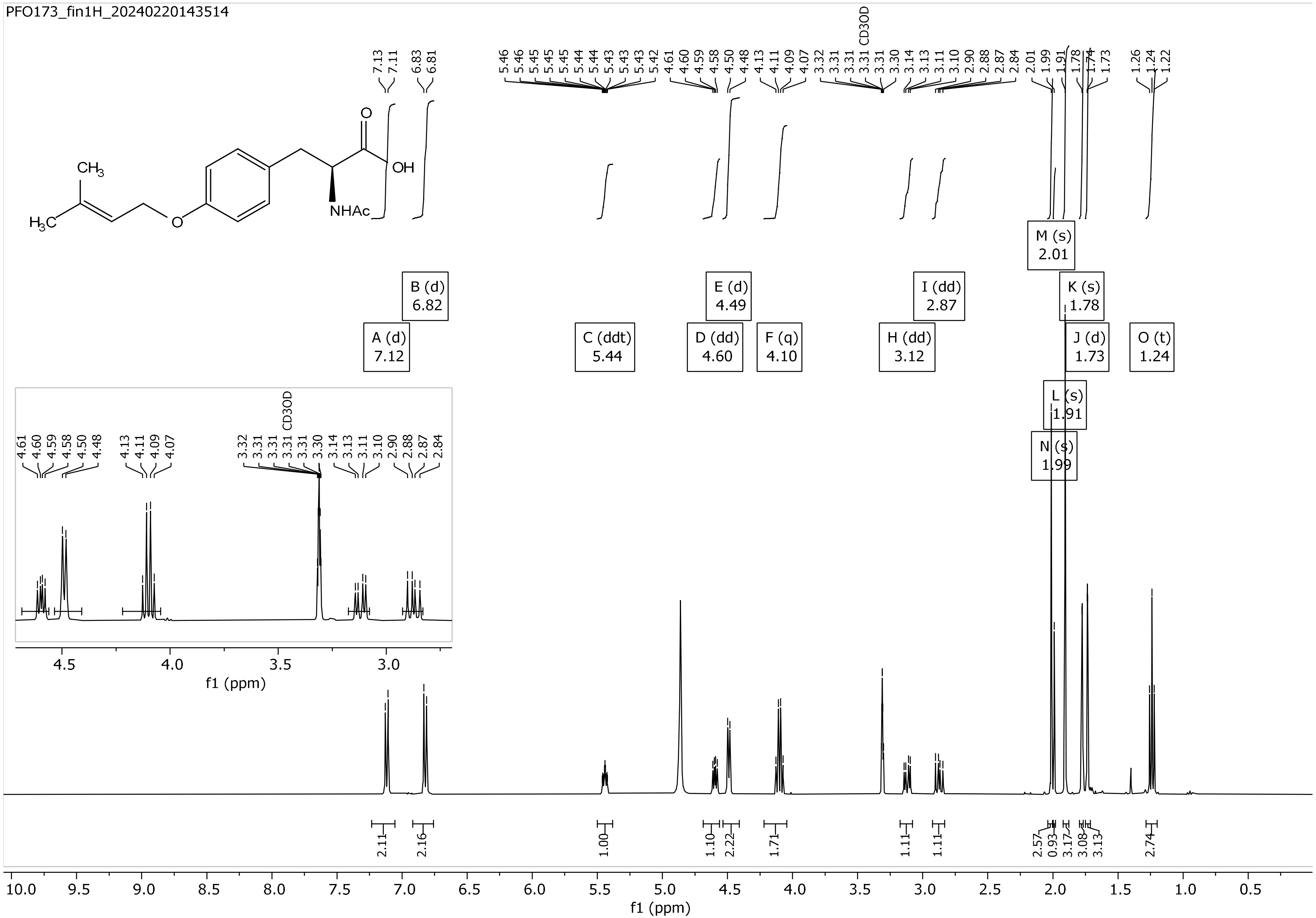

Figure S8. [1]**H NMR spectrum (400 Mhz) of compound PFO173: 4-O-dimethylallyl-N-acetyl-L-tyrosine, in CD_3OD.**

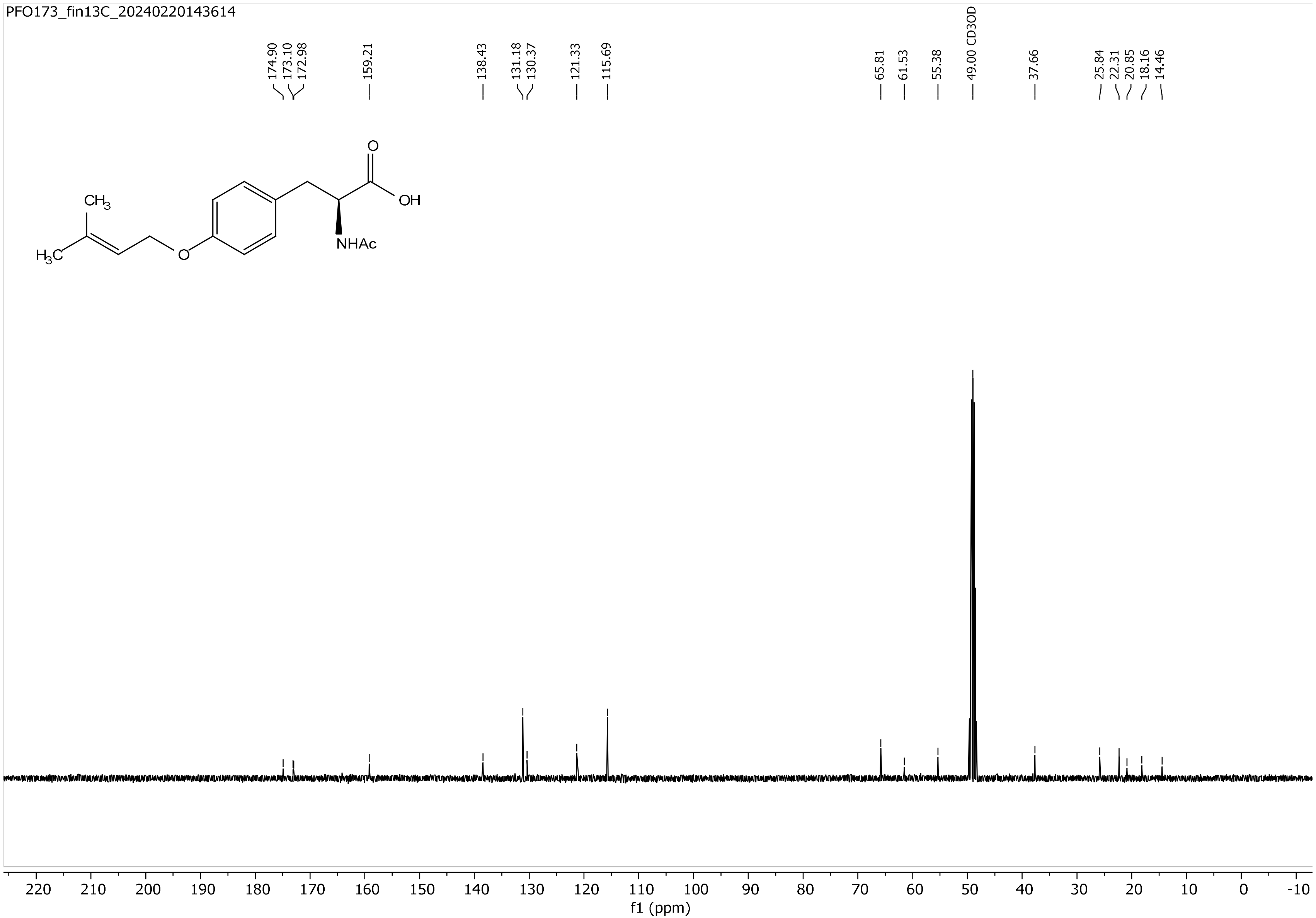

Figure S9. **^{13}C NMR spectrum (101 Mhz) of compound PFO173: 4-O-dimethylallyl-N-acetyl-L-tyrosine, in CD_3OD.**

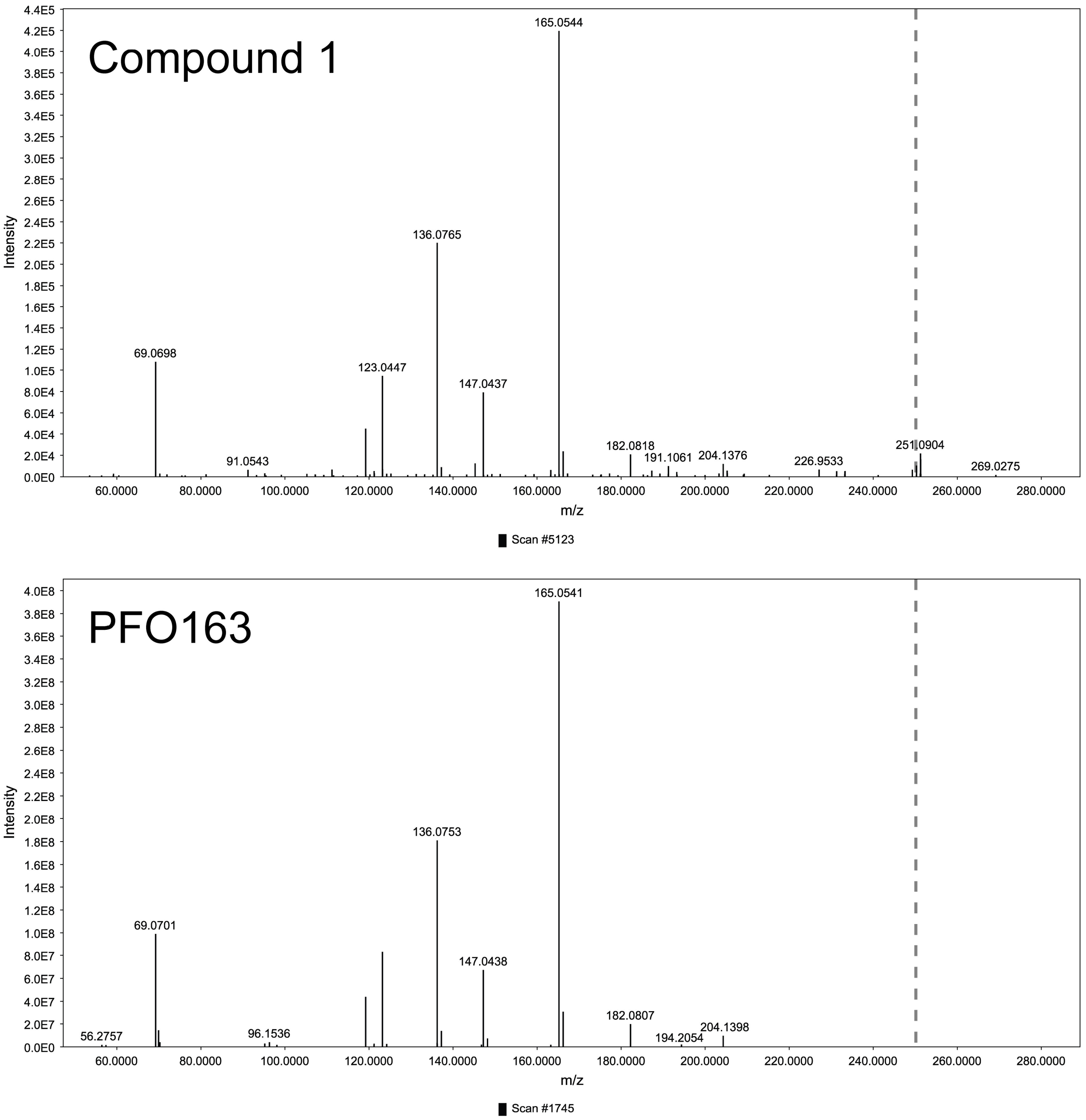

Figure S10. Comparison of MS2 spectra of compound 1 from the extract of the *Ri* DMATS overexpression strain and its chemically-synthesized reference compound, PFO163. Precursor *m/z* value is indicated by a dashed grey line.

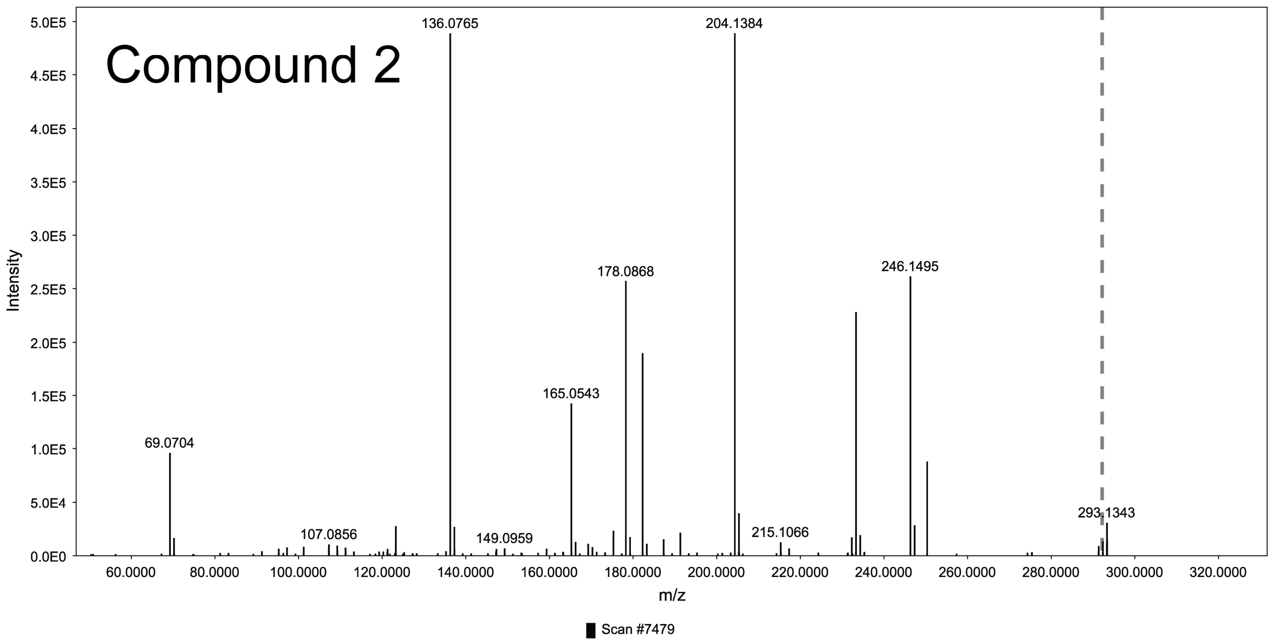

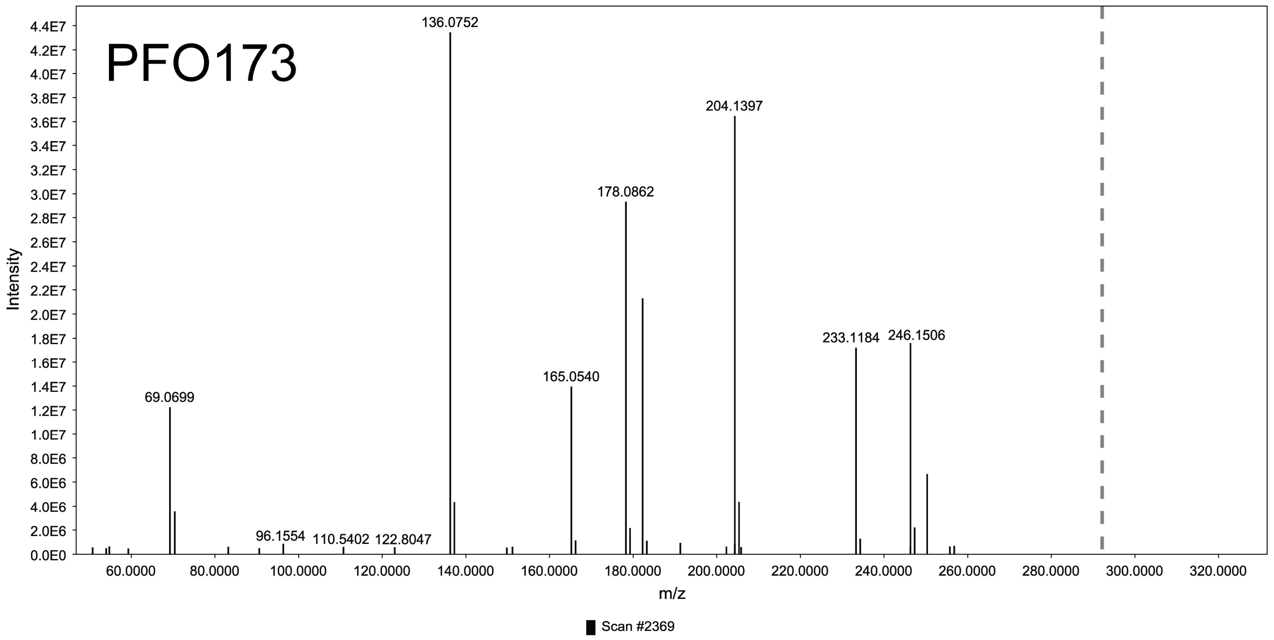

Figure S11. Comparison of MS2 spectra of compound 2 from the extract of the *Ri* DMATS overexpression strain and its chemically-synthesized reference compound, PFO173. Precursor *m/z* value is indicated by a dashed grey line.

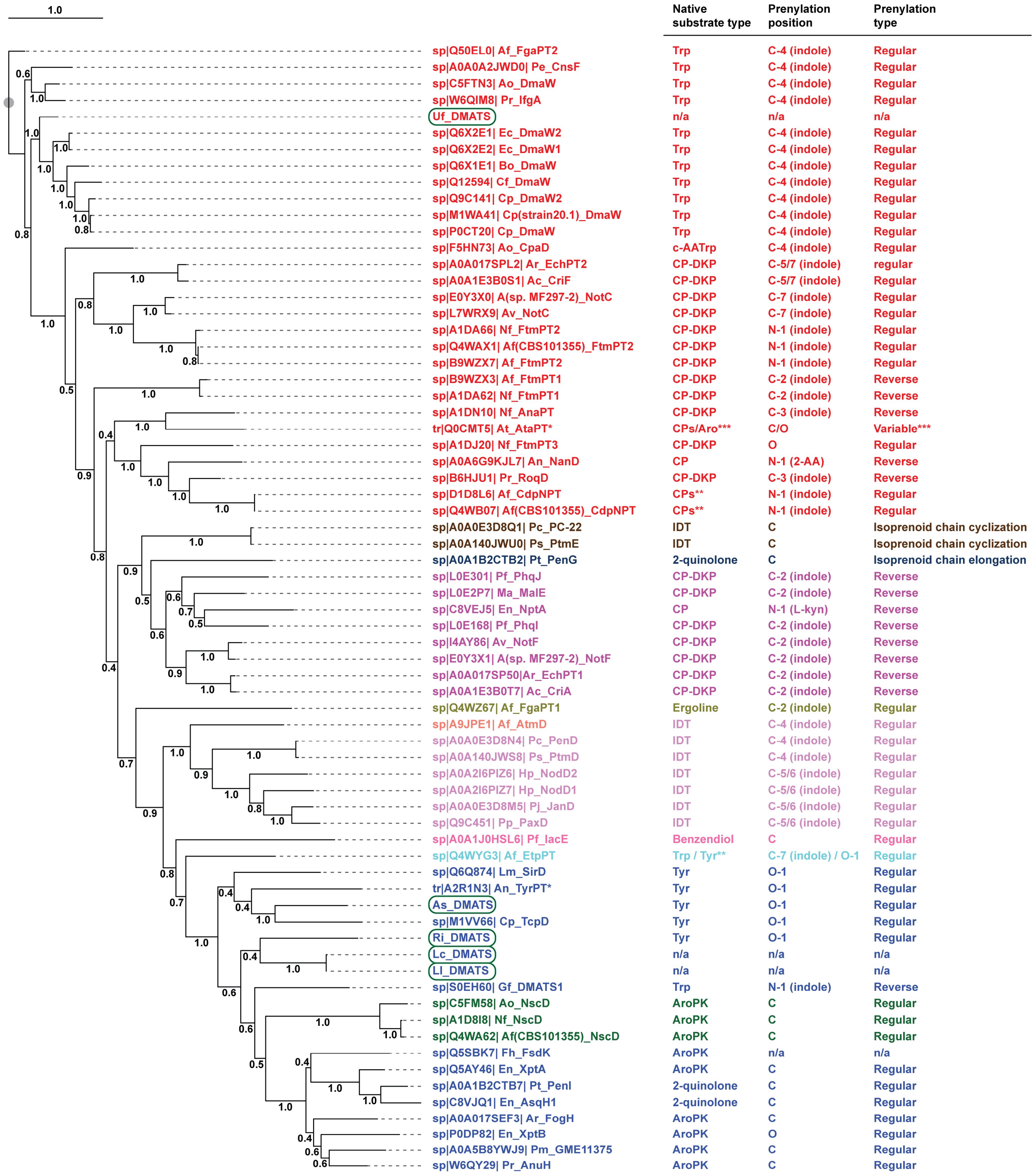

	Native substrate type	Prenylation position	Prenylation type
sp|Q50EL0| Af_FgaPT2	Trp	C-4 (indole)	Regular
sp|A0A0A2JWD0| Pe_CnsF	Trp	C-4 (indole)	Regular
sp|C5FTN3| Ao_DmaW	Trp	C-4 (indole)	Regular
sp|W6QIM8| Pr_IfgA	Trp	C-4 (indole)	Regular
Uf_DMATS	n/a	n/a	n/a
sp|Q6X2E1| Ec_DmaW2	Trp	C-4 (indole)	Regular
sp|Q6X2E2| Ec_DmaW1	Trp	C-4 (indole)	Regular
sp|Q6X1E1| Bo_DmaW	Trp	C-4 (indole)	Regular
sp|Q12594| Cf_DmaW	Trp	C-4 (indole)	Regular
sp|Q9C141| Cp_DmaW2	Trp	C-4 (indole)	Regular
sp|M1WA41| Cp(strain20.1)_DmaW	Trp	C-4 (indole)	Regular
sp|P0CT20| Cp_DmaW	Trp	C-4 (indole)	Regular
sp|F5HN73| Ao_CpaD	c-AATrp	C-4 (indole)	Regular
sp|A0A017SPL2| Ar_EchPT2	CP-DKP	C-5/7 (indole)	regular
sp|A0A1E3B0S1| Ac_CriF	CP-DKP	C-5/7 (indole)	Regular
sp|E0Y3X0| A(sp. MF297-2)_NotC	CP-DKP	C-7 (indole)	Regular
sp|L7WRX9| Av_NotC	CP-DKP	C-7 (indole)	Regular
sp|A1DA66| Nf_FtmPT2	CP-DKP	N-1 (indole)	Regular
sp|Q4WAX1| Af(CBS101355)_FtmPT2	CP-DKP	N-1 (indole)	Regular
sp|B9WZX7| Af_FtmPT2	CP-DKP	N-1 (indole)	Regular
sp|B9WZX3| Af_FtmPT1	CP-DKP	C-2 (indole)	Reverse
sp|A1DA62| Nf_FtmPT1	CP-DKP	C-2 (indole)	Reverse
sp|A1DN10| Nf_AnaPT	CP-DKP	C-3 (indole)	Reverse
tr|Q0CMT5| At_AtaPT*	CPs/Aro***	C/O	Variable***
sp|A1DJ20| Nf_FtmPT3	CP-DKP	O	Regular
sp|A0A6G9KJL7| An_NanD	CP	N-1 (2-AA)	Reverse
sp|B6HJU1| Pr_RoqD	CP-DKP	C-3 (indole)	Reverse
sp|D1D8L6| Af_CdpNPT	CPs**	N-1 (indole)	Regular
sp|Q4WB07| Af(CBS101355)_CdpNPT	CPs**	N-1 (indole)	Regular
sp|A0A0E3D8Q1| Pc_PC-22	IDT	C	Isoprenoid chain cyclization
sp|A0A140JWU0| Ps_PtmE	IDT	C	Isoprenoid chain cyclization
sp|A0A1B2CTB2| Pt_PenG	2-quinolone	C	Isoprenoid chain elongation
sp|L0E301| Pf_PhqJ	CP-DKP	C-2 (indole)	Reverse
sp|L0E2P7| Ma_MalE	CP-DKP	C-2 (indole)	Reverse
sp|C8VEJ5| En_NptA	CP	N-1 (L-kyn)	Reverse
sp|L0E168| Pf_PhqI	CP-DKP	C-2 (indole)	Reverse
sp|I4AY86| Av_NotF	CP-DKP	C-2 (indole)	Reverse
sp|E0Y3X1| A(sp. MF297-2)_NotF	CP-DKP	C-2 (indole)	Reverse
sp|A0A017SP50|Ar_EchPT1	CP-DKP	C-2 (indole)	Reverse
sp|A0A1E3B0T7| Ac_CriA	CP-DKP	C-2 (indole)	Reverse
sp|Q4WZ67| Af_FgaPT1	Ergoline	C-2 (indole)	Regular
sp|A9JPE1| Af_AtmD	IDT	C-4 (indole)	Regular
sp|A0A0E3D8N4| Pc_PenD	IDT	C-4 (indole)	Regular
sp|A0A140JWS8| Ps_PtmD	IDT	C-4 (indole)	Regular
sp|A0A2I6PIZ6| Hp_NodD2	IDT	C-5/6 (indole)	Regular
sp|A0A2I6PIZ7| Hp_NodD1	IDT	C-5/6 (indole)	Regular
sp|A0A0E3D8M5| Pj_JanD	IDT	C-5/6 (indole)	Regular
sp|Q9C451| Pp_PaxD	IDT	C-5/6 (indole)	Regular
sp|A0A1J0HSL6| Pf_IacE	Benzendiol	C	Regular
sp|Q4WYG3| Af_EtpPT	Trp / Tyr**	C-7 (indole) / O-1	Regular
sp|Q6Q874| Lm_SirD	Tyr	O-1	Regular
tr|A2R1N3| An_TyrPT*	Tyr	O-1	Regular
As_DMATS	Tyr	O-1	Regular
sp|M1VV66| Cp_TcpD	Tyr	O-1	Regular
Ri_DMATS	Tyr	O-1	Regular
Lc_DMATS	n/a	n/a	n/a
Ll_DMATS	n/a	n/a	n/a
sp|S0EH60| Gf_DMATS1	Trp	N-1 (indole)	Reverse
sp|C5FM58| Ao_NscD	AroPK	C	Regular
sp|A1D8I8| Nf_NscD	AroPK	C	Regular
sp|Q4WA62| Af(CBS101355)_NscD	AroPK	C	Regular
sp|Q5SBK7| Fh_FsdK	AroPK	n/a	n/a
sp|Q5AY46| En_XptA	AroPK	C	Regular
sp|A0A1B2CTB7| Pt_PenI	2-quinolone	C	Regular
sp|C8VJQ1| En_AsqH1	2-quinolone	C	Regular
sp|A0A017SEF3| Ar_FogH	AroPK	C	Regular
sp|P0DP82| En_XptB	AroPK	O	Regular
sp|A0A5B8YWJ9| Pm_GME11375	AroPK	C	Regular
sp|W6QY29| Pr_AnuH	AroPK	C	Regular

Figure S12. Maximum-likelihood phylogenetic tree of characterized DMATS-type PTs and lichen DMATS (green circles). *Uf* DMATS is included as the only lichen DMATS-type PT that clustered with prototype DMATS sequences. The tree was arbitrarily rooted in the *Af* FgaPT2 node. Substrates are abbreviated as follows. Trp: tryptophan; Tyr: tyrosine; c-aaTrp: cyclo-acetoacetyl-L-tryptophan; CP-DKP: cyclic peptide-diketopiperazine; CP: cyclic peptide; IDT: indole diterpene; Aro: aromatics (general); AroPK: aromatic polyketides. Entries are colored based on their respective clusters in Figure 3. Bootstrap values are given for each node (1000 replicates). *These enzymes are not in the SwissProt database, but they have been characterized experimentally. **Unnatural substrates tested in vitro. ****At* AtaPT was only tested *in vitro* but showed an incredible range of acceptor and donor substrate[6].

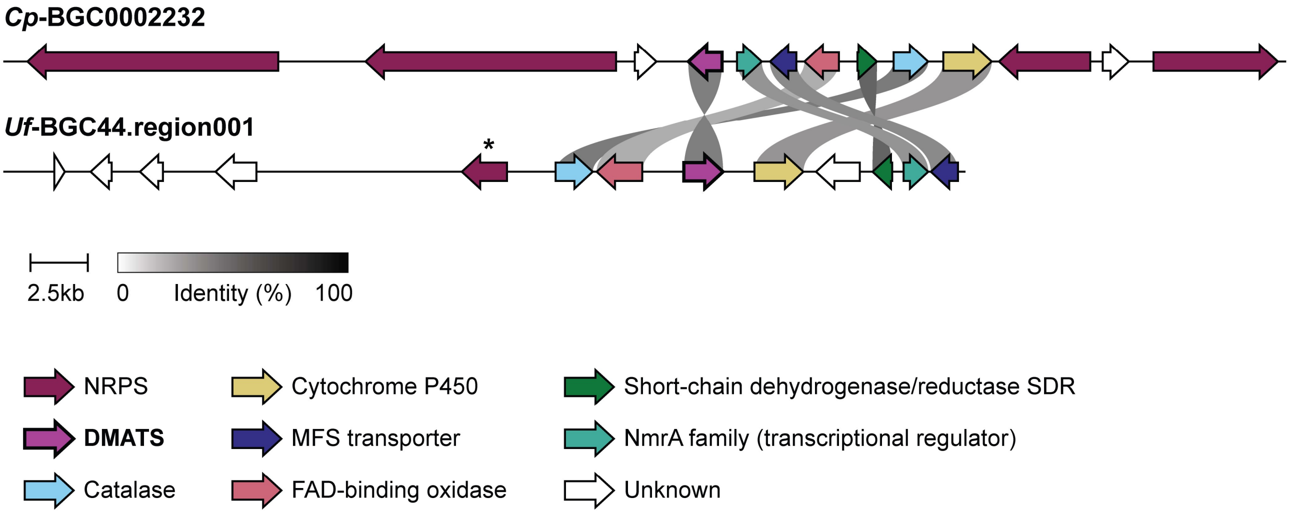

Figure S13. Comparison of ergotamine BGC from *Claviceps purpurea* (MIBiG accession no. BGC0002232) to NRPS/indole BGC 44.1 from *Usnea florida* (scaffold 44, 300,249 – 342,216 nt). The genes are colored based on their known or putative function. Identity between individual genes is highlighted according to provided grayscale gradient. *The putative NRPS on the BGC from *U. florida* appears to be truncated, but this might be due to inaccurate sequencing and/or genome annotation. This is also suggested by the large stretch (~9 kb) of unannotated DNA immediately downstream of the annotated gene.

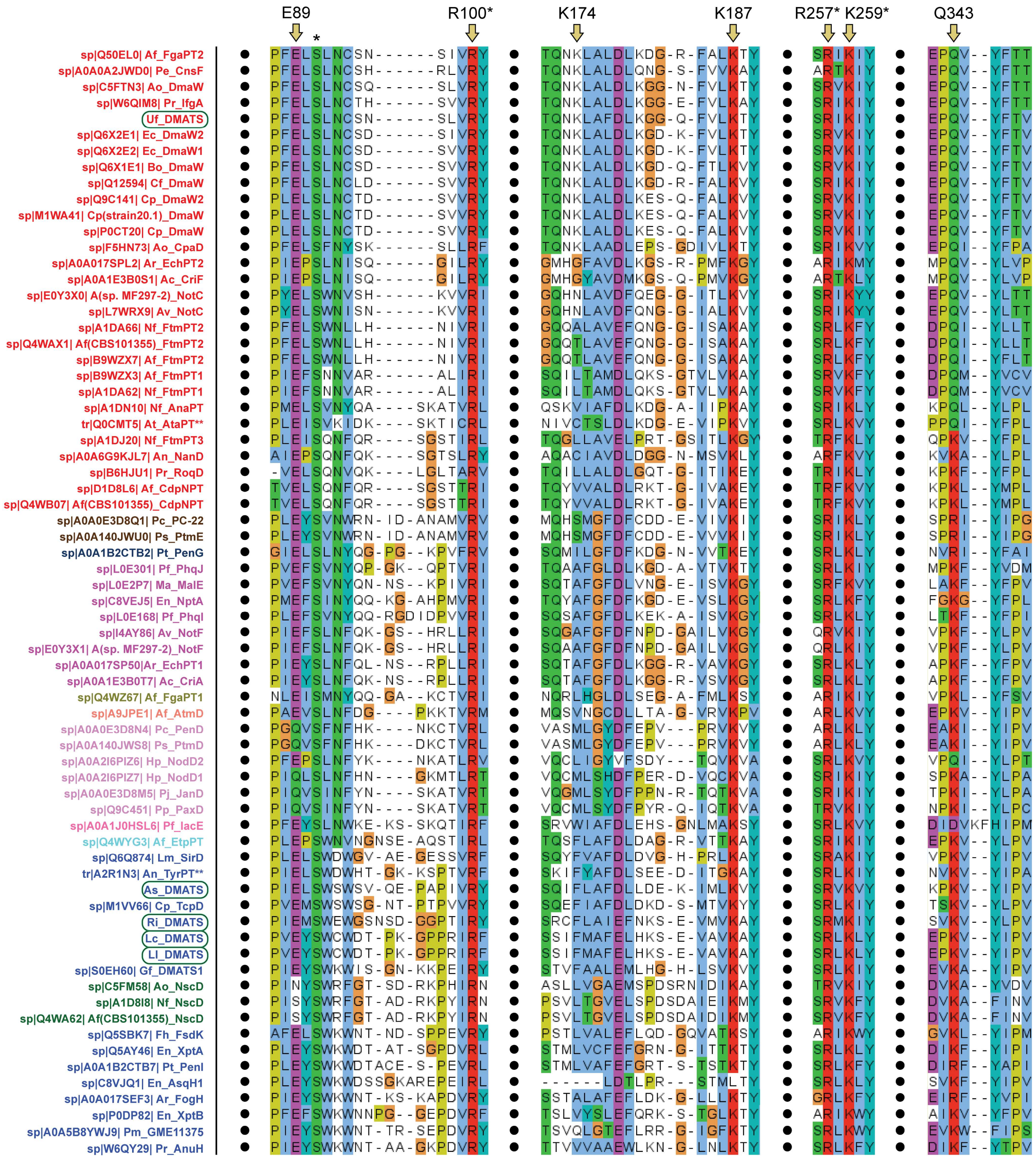

Figure S14. Multiple sequence alignment of characterized DMATS-type PTs and lichen DMATS (green circles). *Uf* DMATS is included as the only lichen DMATS-type PT that clustered with prototype DMATS sequences. The alignment is trimmed at relevant positions where key catalytic residues are found; the full alignment is available as additional file 2. Residues that are 100% conserved are highlighted by a star. Residues that are important for binding of substrates and catalysis are labeled and indicated by yellow arrows. **These enzymes are not in the SwissProt database, but they have been characterized experimentally.

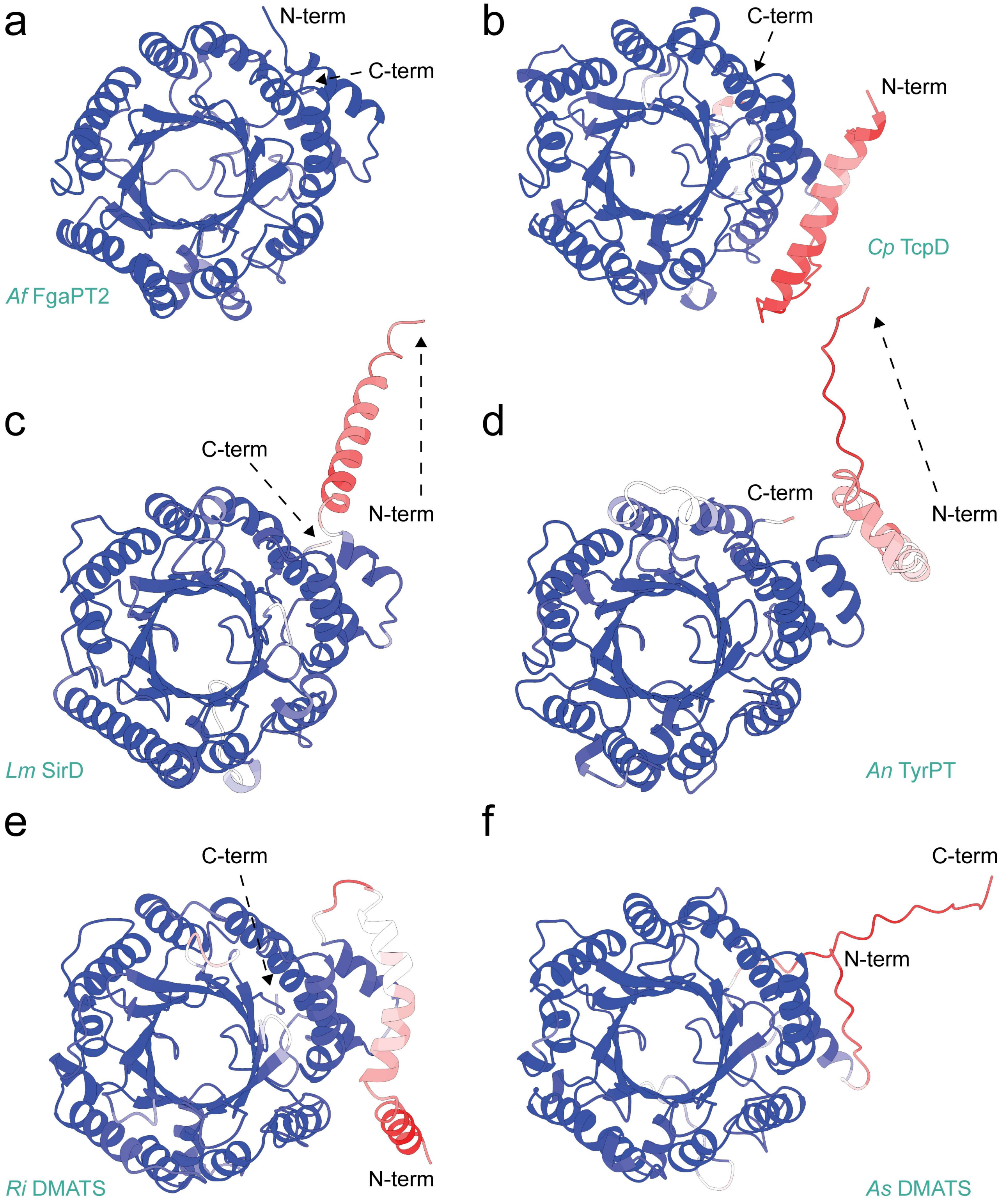

Figure S15. Comparison of structural models of 4-O-dimethylallyltyrosine synthases (AlphaFold predictions) to the crystal structure of FgaPT2 (PDB ID: 3I4X). The models are colored based on AlphaFold per-residue confidence measure pLDDT (higher is better, blue), while FgaPT2 is colored based on B-factor (lower is better, blue).

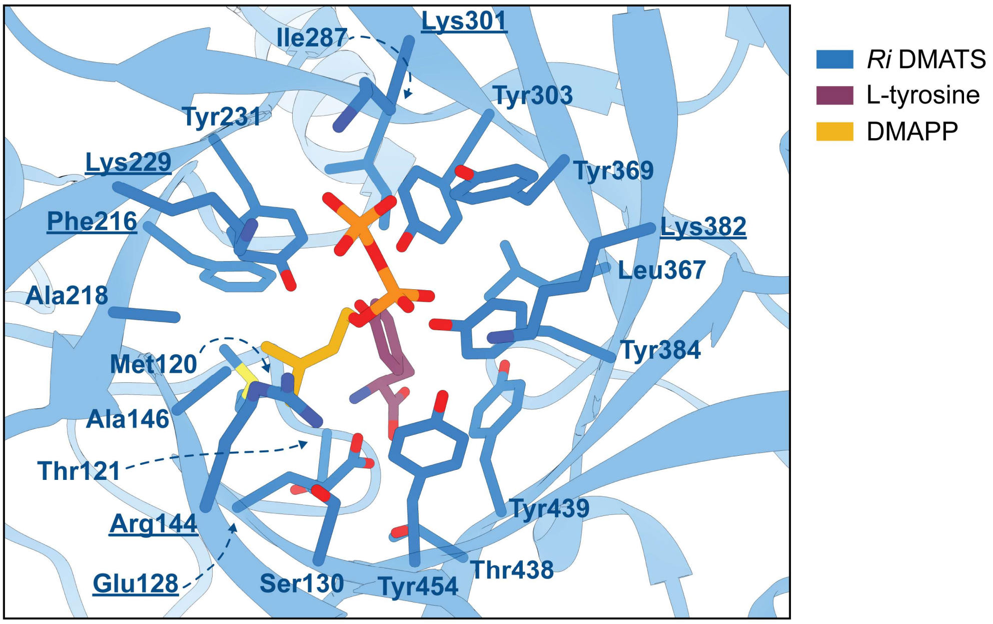

Ri DMATS active site

Figure S16. Active site of *Ri* DMATS with substrates DMAPP and L-Tyr docked within the pocket. Conserved positively charged residues Arg144, Lys229, and Lys301 interact with the pyrophosphate group as expected. The 4-OH group of tyrosine is correctly oriented to bind the dimethylallyl donor. The resulting intermediate cation could be deprotonated by a molecule of water or by Lys382, which has the ε-amino group positioned in the vicinity of the newly formed bond. Stick models colored by element: oxygen – red, nitrogen – dark blue, sulfur – light yellow, phosphate – orange, carbon – light blue (*Ri* DMATS)/ purple (L-tyrosine)/ dark yellow (DMAPP).

References

(1) McDonald, T. R.; Mueller, O.; Dietrich, F. S.; Lutzoni, F. High-Throughput Genome Sequencing of Lichenizing Fungi to Assess Gene Loss in the Ammonium Transporter/Ammonia Permease Gene Family. *BMC Genomics* **2013**, *14* (1), 225. https://doi.org/10.1186/1471-2164-14-225.

(2) Ahrendt, S. R.; Mondo, S. J.; Haridas, S.; Grigoriev, I. V. MycoCosm, the JGI's Fungal Genome Portal for Comparative Genomic and Multiomics Data Analyses. In *Martin, F., Uroz, S. (eds) Microbial Environmental Genomics (MEG). Methods in Molecular Biology, vol 2605. Humana, New York, NY.*; 2023; pp 271–291. https://doi.org/10.1007/978-1-0716-2871-3_14.

(3) McKenzie, S. K.; Walston, R. F.; Allen, J. L. Complete, High-Quality Genomes from Long-Read Metagenomic Sequencing of Two Wolf Lichen Thalli Reveals Enigmatic Genome Architecture. *Genomics* **2020**, *112* (5), 3150–3156. https://doi.org/10.1016/j.ygeno.2020.06.006.

(4) Hackl, T.; Ankenbrand, M.; van Adrichem, B. Gggenomes: A Grammar of Graphics for Comparative Genomics. R Package Version 1.0.0. **2024**.

(5) Blin, K.; Shaw, S.; Augustijn, H. E.; Reitz, Z. L.; Biermann, F.; Alanjary, M.; Fetter, A.; Terlouw, B. R.; Metcalf, W. W.; Helfrich, E. J. N.; van Wezel, G. P.; Medema, M. H.; Weber, T. AntiSMASH 7.0: New and Improved Predictions for Detection, Regulation, Chemical Structures and Visualisation. *Nucleic Acids Res.* **2023**, *51* (W1), W46–W50. https://doi.org/10.1093/nar/gkad344.

(6) Chen, R.; Gao, B.; Liu, X.; Ruan, F.; Zhang, Y.; Lou, J.; Feng, K.; Wunsch, C.; Li, S.-M.; Dai, J.; Sun, F. Molecular Insights into the Enzyme Promiscuity of an Aromatic Prenyltransferase. *Nat. Chem. Biol.* **2017**, *13* (2), 226–234. https://doi.org/10.1038/nchembio.2263.
